# Home Health Aides Caring for Adults With Heart Failure: A Pilot Randomized Clinical Trial

**DOI:** 10.1001/jamanetworkopen.2025.48121

**Published:** 2025-11-10

**Authors:** Madeline R. Sterling, Cisco G. Espinosa, Sasha Vergez, Margaret V. McDonald, Joanna Ringel, Jonathan N. Tobin, Samprit Banerjee, Nicola Dell, Lisa M. Kern, Monika M. Safford

**Affiliations:** 1 Weill Cornell Medicine, New York, New York; 2 Center for Home Care Policy & Research, VNS Health, New York, New York; 3 Clinical Directors Network, New York, New York; 4 Rockefeller University, New York, New York; 5 Cornell Tech, New York, New York

## Abstract

**Importance:**

Home health aides (HHAs) frequently care for adults with heart failure (HF), but many lack HF training, confidence with HF caregiving, and cannot reach their nurse supervisors by telephone when they need guidance. This may have negative consequences for HHAs and patients.

**Objective:**

To examine the effectiveness of an education- and communication-based intervention among HHAs caring for patients with HF.

**Design, Setting, and Participants:**

This 2-group pilot randomized clinical trial was conducted in partnership with a large home care agency in New York, New York, from May 2022 to May 2024. HHAs caring for a patient with HF participated. Outcomes were ascertained on an intent-to-treat basis at baseline, mid-study (45 days after the training course), and 90 days.

**Interventions:**

The enhanced usual care (EUC) group received HF training, and the intervention group received HF training plus a mobile health application that allowed HHAs to message nurses.

**Main Outcomes and Measures:**

Co–primary outcomes were HF knowledge (assessed using the Dutch HF Knowledge Scale [DHFKS]; range 0-15; higher score indicates greater knowledge) and HF caregiver self-efficacy (assessed using the Caregiver Contribution to Self-Care in HF Index; range, 0-100; higher score indicates greater efficacy). The secondary outcome was self-reported preventable 911 calls. Exploratory outcomes included patient emergency department (ED) visits and hospitalizations. Mixed-effects models were used to compare trajectories of outcomes between and within study groups.

**Results:**

A total of 102 HHAs (mean [SD] age, 54 [10.5] years; 98 [96.1%] female) were assessed, including 50 in the EUC group and 52 in the intervention group. Overall, 62 HHAs (62.0%) were Black, 1 HHA (1.0%) was American Indian or Alaska Native, 7 HHAs (7.0%) were Asian, 9 HHAs (9.0%) were White, and 21 HHAs (21.0%) identified as other race; 27 HHAs (27.0%) were Hispanic. Within the intervention group, DHFKS scores improved at 90 days, from a median (IQR) score of 6.1 (5.5-6.7) points at baseline to 7.7 (7.0-8.4) points at 90 days (*P* = .02); however the change did not differ between groups. Across both groups, HHAs with the lowest baseline DHFKS and self-efficacy had the greatest increases at 90 days (median [IQR] change: DHFKS, 1.45 [0.84-2.04] points; self-efficacy, 8.06 [4.42-11.71] points). At 90 days, there were no statistically significant within-group differences in the proportion of HHAs reporting preventable 911 calls group (intervention: 0.51 [95% CI, 0.37-0.64] at baseline vs. 0.34 [95% CI, 0.2-0.49] at 90 days; *P* = .06; EUC: 0.42 [95% CI, 0.28-0.56] at baseline vs 0.54 [95% CI, 0.38-0.70] at 90 days; *P* = .21), but the difference between groups was statistically significant (*P* = .04). This pilot study was not powered for patient-level outcomes, so the risk of ED visits for patients of intervention HHAs (incidence rate ratio, 0.56 [95% CI, 0.25-1.28]; *P* = .17) should be considered exploratory.

**Conclusions and Relevance:**

In this randomized clinical trial of HHAs caring for patients with HF, HF training improved HHAs’ knowledge and self-efficacy, with greatest gains among those with the lowest baseline scores. The ability to message nurses was associated with fewer preventable 911 calls among HHAs in the intervention group. These findings can inform the design of a large-scale trial to better support and integrate HHAs providing HF care.

**Trial Registration:**

ClinicalTrials.gov Identifier: NCT04239911

## Key Points

**Question:**

Can an education and communication-based intervention improve knowledge and caregiving self-efficacy among home health aides and attendants (HHAs) caring for adults with heart failure (HF)?

**Findings:**

In this pilot randomized clinical trial including 102 agency-employed HHAs randomized to training alone or in addition to an application that allowed HHAs to exchange text messages with nurse supervisors, training improved HHAs’ HF knowledge and HF caregiving self-efficacy. The addition of the application did not improve these primary outcomes, but it significantly reduced HHAs’ self-reported preventable 911 calls, a secondary outcome.

**Meaning:**

These results support a future RCT that will test the effectiveness of the intervention on outcomes for both HHAs and their patients with HF.

## Teaser

This pilot randomized clinical trial examines the effectiveness of an education- and communication-based intervention among home health aides caring for patients with heart failure.

## Introduction

Heart failure (HF) affects more than 6 million people in the US, with prevalence increasing as the population ages. HF is associated with high morbidity and mortality and poor quality of life.^1,2^ Additionally, HF is responsible for 1 million hospitalizations per year, with 1 of 4 hospitalizations resulting in a readmission within 30 days.^1,2,3,4,5^ Hospitalizations and readmissions, although sometimes medically necessary, can be devastating for patients and families, and are costly for the health care system.^3,6^

While numerous interventions have aimed to support patients with HF in the home and improve outcomes, few have focused on home health aides and attendants (HHAs), a growing health care workforce increasingly involved in HF care. Prior studies have found that HHAs are involved in key aspects of HF care, including monitoring for signs and symptoms of the disease, preparing meals, and reminding patients to take medication.^7,8^ However, a survey of agency-employed HHAs in New York, New York, found that 65% lacked HF training and only 40% felt confident providing HF care.^7,9,10,11,12^ Additionally, many were unable to reach their supervisors at their home care agency (eg, a nurse or a clinical coordinator) by telephone when they had questions about their patient or needed clinical support.^7,13,14^ This is problematic in terms of keeping patients with HF at home, as many HHAs report that their 911 calls could have been prevented if they had been able to reach their nurse supervisor or their patients’ physician.^15^

To address this gap, we previously developed and piloted an HF training course for HHAs that improved their HF knowledge and caregiving confidence.^16^ However, this was tested using a single-group design and among HHAs at large, not specifically those caring for patients with HF. We also partnered with a mobile health (mHealth) application (app) that HHAs employed at certain agencies use (for training and shift booking) to offer them a new chat feature, which allowed them to message an office-based nurse case manager in real time.^13,17^ Although we gathered feedback from HHAs and agency staff on this chat feature, it had not been piloted among HHAs while they provide care to patients with HF.

To that end, the goal of this 2-group pilot randomized clinical trial (RCT) was to assess the feasibility, acceptability, and preliminary effectiveness of this education- and communication-based intervention among agency-employed HHAs caring for community-dwelling adults with HF. With respect to effectiveness, which is the focus of this study, we aimed to assess the effectiveness of the intervention on HHAs’ HF knowledge and HF caregiving self-efficacy (co–primary outcomes) and preventable 911 calls (secondary outcome) and, in an exploratory fashion, examine the effectiveness of the intervention on the outcomes among patients for whom the HHAs were caring, specifically emergency department (ED) visits and hospitalizations. We hypothesized that there would be statistically significant differences with respect to the primary and secondary outcomes between the study groups (enhanced usual care [EUC] vs intervention). Although not powered to detect differences in patient outcomes, we hypothesized that patients with HHAs in the intervention group would have fewer adverse outcomes (ED visits) than those in the control group.

## Methods

### Overview and Study Design

This RCT was approved by the VNS Health and the Weill Cornell Medicine institutional review boards. All HHAs provided electronic written informed consent. This study is reported following the Consolidated Standards of Reporting Trials (CONSORT) reporting guideline. The protocol for this study was previously published^18^ and is available in Supplement 1. Briefly, we conducted this study in partnership with VNS Health, a large, nonprofit organization that provides home care, population management, community health services, and hospice care to adults living in New York, New York. The study was conducted among HHAs employed within the licensed home care service agency (LHCSA) of the organization, which employs more than 6500 HHAs who provide personal care and health-related assistance to patients for extended periods of time in the home.^19^

In this single-site parallel-group pilot RCT, HHAs were randomized to either the intervention group or EUC group. All participants were in the trial for 90 days. Data from HHAs were collected from participants at baseline (0 days), midstudy (45 days), and follow-up (90 days). Recruitment began in May 2022 and ended in May 2024.

### Participants and Eligibility Criteria

#### HHAs

HHAs were the main (or primary) participants of the trial and were eligible if they spoke English or Spanish, were employed by the participating LHCSA, had a smartphone or email address, and provided care (during the study) to a patient with HF enrolled in a managed long-term health care plan, not including hospice care. HF was defined using *International Statistical Classification of Diseases and Related Health Problems, Tenth Revision* (*ICD-10*) codes I09.9, I11.0, I13.0, I13.2, I25.5, I42.0, I42.5 - I42.9, I43.x, I50.x, and P29.0. Recruitment procedures for HHAs are described elsewhere.^18^ All HHAs were compensated $100 total for participating ($25 per data collection point). To recruit HHAs caring for patients with HF, this study required access to patient records, granted through a Health Insurance Portability and Accountability Act (HIPAA) waiver of authorization. The intervention did not involve direct interaction with patients.

#### Nurses

Four nurses were recruited to deliver the intervention in the study (ie, respond to text messages of intervention HHAs). Nurses were office-based and provided clinical oversight of patients with HF. All nurses provided verbal consent and were compensated $100 for participating.

### Randomization

Once enrolled, HHAs were oriented to the study, completed a baseline survey, and their smartphone was registered by the study team. Participants were enrolled by research staff in person using a sequentially numbered list. Randomization to a study group was created using a block randomization design (blocks of 4 and 6 using the R package blockrand [R Project for Statistical Computing]) by the statistical analysts (J.R. and S.B.). As a behavioral intervention and pilot RCT, participants were not blinded to their study group, nor were research assistants who onboarded them. However, separate research team members were selected and blinded to outcome ascertainment and solely conducted analysis.

### EUC Group

Participants in the EUC group received a virtual HF training course; the development and piloting of the course has been previously described.^16,20^ Briefly, the 1.5-hour course was developed to meet HHAs’ educational needs while aligning with their scope of care. The course, delivered via video conferencing software (Zoom Communications), provided HHAs with an overview of HF, including its signs and symptoms, with most of the education occurring through facilitated case-based learning. Clinical vignettes review symptom recognition and monitoring, lifestyle behaviors, medication adherence, and triaging emergencies in the home.^16^ The synchronous course was delivered to small groups of HHAs at a time by a trained instructor (including clinicians, research assistants, and agency staff). In line with HHAs’ desire to access course content on-demand, we created a website so they can access HF-related content for free and at any time.^21^

### Intervention Group

Participants in the intervention group received a virtual HF training course and the mHealth app chat tool (via CareConnect). The chat tool enables HHAs to message the study nurses in a HIPAA-adherent manner when caring for their patient with HF. Chats were encouraged to be broad in nature, including clinical questions (eg, patient symptoms), advice and guidance on personal and medical care, and general updates.

At the start of the study (after randomization and orientation), the nurse assigned to the participant (HHA) was asked to initiate a test chat to ensure a working connection in CareConnect. HHAs were instructed to message back to be able to start chatting. Study staff sent automatic Short Message Service text-based reminders (outside of CareConnect) to reinforce this behavior change (of chatting via the app) at the beginning of each HHAs’ study period. During the study, HHAs were instructed to use the chat (as needed) without any minimum use requirements.

### Data Collection

In line with the goals of this feasibility pilot RCT, we collected data using a multimodal approach from a variety of participants. First, we used surveys comprised of novel and validated measures to gather sociodemographic data and assess effectiveness (of main and secondary outcomes) among HHAs. Surveys were administered through REDCap software (Vanderbilt University), a secure data management system. Second, we used study staff team observations and fidelity checklists to assess aspects of feasibility and adoption. Third, we conducted semistructured qualitative interviews with HHAs, nurses, study staff, and patients to assess feasibility and acceptability. Fourth, we examined app message content and frequency between the HHAs and nurses (intervention group) to assess engagement with the intervention.

### Outcomes

Feasibility and acceptability will be reported elsewhere. The focus of this analysis is effectiveness related. In line with presenting effectiveness data, we include limited engagement data as they pertain to the intervention.

### Primary Outcomes

This study had 2 co–primary outcomes, HF knowledge and HF caregiving self-efficacy. HF knowledge was assessed with the Dutch HF Knowledge Scale (DHFKS), a 15-item scale that measures HF knowledge, including treatments and symptom recognition. Scores range from 0 to 15 points, with higher scores indicating greater knowledge.^22^ This scale was previously used among HHAs.^16^

HF caregiving self-efficacy was assessed within the Caregiver Contribution to Self-Care in HF Index (CC-SCHFI).^10,23,24,25^ The CC-SCHFI is a 22-item scale that measures caregivers’ contributions to HF maintenance, HF management, and HF caregiving self-efficacy (confidence). Each subscale is scored independently. The third subscale, also known as the 10-item HF Caregiving Self-Efficacy Scale, was the outcome of interest. HF caregiving self-efficacy scores range from 0 to 100 points, with higher scores indicating greater self-efficacy. This scale has previously been validated (Cronbach α = .94) by our team among HHAs (factor score determinacy coefficient, 0.80-0.87).^10^ For this study, we only examined effectiveness using the HF Caregiving Self-Efficacy Scale.

### Secondary Outcomes

Preventable 911 calls were a secondary outcome. This was assessed with 2 close-ended questions that we previously used among HHAs.^15^ HHAs were asked to reflect on caring for a HF patient and asked: “I have called 911 when that could have been prevented if I had been able to reach my supervisor/the nurse” and “I have called 911 when that could have been prevented if my supervisor and I had been able to reach the client’s doctor.”^15^ Responses for both included a 7-point Likert scale, with 1 indicating very strongly disagree; 2, strongly disagree; 3, disagree; 4, neutral; 5, agree; 6, strongly agree; and 7, very strongly agree. Both questions were dichotomized into agree, strongly agree, and very strongly agree vs all other responses.

### Covariates

In addition to primary and secondary outcomes, self-reported demographic characteristics, including age, sex, race (including Asian, Black, White, and other [with free-text responses, including Black/Puerto Rican, Latino, Hispanic, Caribbean, Dominican Republic, and Mestizo, or not specified]), Hispanic or Latinx ethnicity, educational level, and employment history, were collected among HHAs. Employment-related data, as well as prior experience with HF training and caregiving and contributions to HF care, were assessed with the CC-SCHFI scales. The Caregiver Contribution to Self-Care Maintenance Scale includes 10 items and measures the extent to which caregivers of patients with HF recommend behaviors aimed at maintaining HF stability. The Caregiver Contribution to Self-Care Management Scale includes 6 items and measures caregivers’ responses in dealing with signs and symptoms of HF exacerbation.^23^ Both scores range from 0 and 100 points, with higher scores indicating a greater contribution to HF patient self-care.

### Patient Outcomes

Patient outcomes included all-cause ED visits and hospitalizations at 90 days. These outcomes were ascertained from a combination of data sources: service use records (including dates and reason for service use disruption) from the LHCSA, and health information exchange, which contains hospitalization and ED events. Exclusion criteria selected for patients who had multiple HHAs during the trial or those assigned to HHAs who had multiple patients.

### Intervention Engagement

Among HHAs in the intervention group, engagement with the mHealth app was defined as messaging the nurse back after receiving an initial welcome chat (yes or no). Additional engagement outcomes included HHAs with frequent messages (≥2 messages with a nurse during the study period) and content of messages.

### Statistical Analysis

We used descriptive statistics to characterize the overall study population. Next, we examined differences in the study population by study group using Pearson χ^2^ or Fisher exact tests for categorical variables and Wilcoxon rank-sum tests for continuous variables.

We next aimed to measure change in scores from baseline to postcourse and 90-day assessments in the intervention group compared with the EUC group. We used linear mixed-effects models to compare the trajectory of all outcomes between and within study arms. Data included an observation for each time point (baseline, postcourse, and 90 days). Mixed-effects models included a fixed-effects categorical variable for time point, an indicator for study group, a study group × time point interaction, and participant-specific random intercept. Logistic mixed-effects models were used to assess secondary outcomes with similar fixed and random effects. These logistic mixed-effects models only included 2 time points (baseline and 90 days) as they were not assessed at postcourse.

As an exploratory post hoc analysis, we investigated the trajectory of DHFKS and self-efficacy among participants with low baseline scores, since we hypothesized that these participants might stand to gain the most from the interventions. We used linear mixed-methods models with similar methods to the primary analysis. Rates of health care utilization among patients cared for by HHAs in the EUC vs intervention group were compared using Poisson models.

With respect to mHealth app messages between HHAs and nurses in the intervention arm, we used descriptive statistics to summarize their frequency, and we qualitatively coded the content of the messages using a content-based analysis. Using an inductive coding approach, 2 researchers (including M.R.S.) independently coded the messages to create a codebook, which was then applied to the message logs. The research team then grouped similar codes into categories and ultimately broader themes of message types. As detailed in the trial protocol in Supplement 1, we conducted qualitative interviews to assess the feasibility of the trial and intervention with participants and staff, however the findings from these are reported elsewhere.^18^

Missing data were handled with listwise deletion. For all analyses, 2-sided *P* < .05 was used to determine significance. Data were analyzed from May 29, 2024, to June 23, 2025, using Stata version 18 (StataCorp).

## Results

### Study Population

**Figure 1.**
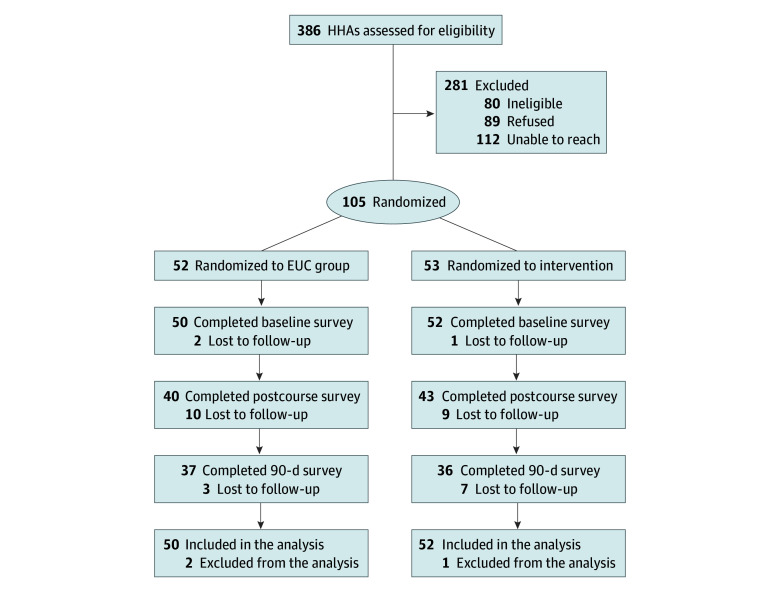
Study Enrollment Flowchart EUC indicates enhanced usual care; HHA, home health aide.

A total of 102 HHAs (mean [SD] age, 54 [10.5] years; 98 [96.1%] female) were included, with 50 HHAs in the EUC group and 52 HHAs in the intervention group. A detailed study flowchart is shown in Figure 1. Baseline characteristics are shown in Table 1, stratified by study group. Overall, 62 HHAs (62.0%) were Black, 1 HHA (1.0%) was American Indian or Alaska Native, 7 HHAs (7.0%) were Asian, 9 HHAs (9.0%) were White, and 21 HHAs (21.0%) identified as other race; 27 HHAs (27.0%) were Hispanic; and 88 HHAs (86.3%) were born outside the US. With respect to primary language spoken, 65 HHAs (63.7%) spoke English and 20 HHAs (19.6%) spoke Spanish. Approximately one-quarter of HHAs (28 [27.5%]) had 0 to 5 years of work experience as an HHA, 31 HHAs (30.4%) had 6 to 10 years, 16 HHAs (15.7%) had 11 to 15 years, 27 HHAs (26.5%) had more than 15 years of experience. Most participants had worked for 1 or 2 other home health agencies in the past (Table 1).

**Table 1.** Home Health Aide Characteristics by Study Group

Characteristic	Participants, No. (%)		
	Total (N = 102)	EUC (n = 50)	Intervention (n = 52)
Sex			
Male	4 (3.9)	2 (4)	2 (4)
Female	98 (96.1)	48 (96)	50 (96)
Age, mean (SD), y (n = 101)	54.0 (10.5)	53.2 (10.9)	54.8 (10.2)
Race (n = 100)			
Black	62 (62.0)	30 (61)	32 (63)
American Indian or Alaska Native	1 (1.0)	1 (2)	0
Asian	7 (7.0)	3 (6)	4 (8)
White	9 (9.0)	6 (12)	3 (6)
Other^a^	21 (21.0)	9 (18)	12 (24)
Hispanic ethnicity (n = 100)	27 (27.0)	14 (29)	13 (25)
Education (n = 101)			
No degree or ≤high school	21 (20.8)	9 (18)	12 (23)
Completed high school or GED	36 (35.6)	18 (37)	18 (35)
Some college	20 (19.8)	12 (24)	8 (15)
College degree	17 (16.8)	8 (16)	9 (17)
Graduate degree	7 (6.9)	2 (4)	5 (10)
Born outside the US	88 (86.3)	45 (90)	43 (83)
Primary language			
English	65 (63.7)	33 (66)	32 (62)
Spanish	20 (19.6)	11 (22)	9 (17)
Other	17 (16.7)	6 (12)	11 (21)
Years as HHA			
0-5	28 (27.5)	13 (26)	15 (29)
6-10	31 (30.4)	14 (28)	17 (33)
11-15	16 (15.7)	9 (18)	7 (13)
>15	27 (26.5)	14 (28)	13 (25)
Home care agencies worked for in the past, No. (n = 100)			
0-1	53 (52.0)	26 (52)	27 (52)
2	33 (32.4)	17 (34)	16 (31)
≥3	16 (15.7)	7 (14)	9 (17)
Years worked at current agency. No. (n = 100)			
0-5	35 (34.7)	16 (32)	19 (37)
6-10	34 (33.7)	16 (32)	18 (35)
11-15	18 (17.8)	10 (20)	8 (16)
>15	14 (13.9)	8 (16)	6 (12)
Patients with HF cared for, No.			
≤5	65 (63.7)	38 (76)	27 (52)
>5	18 (17.6)	6 (12)	12 (23)
Not sure	19 (18.6)	6 (12)	13 (25)
Time spent with patient with HF, h/wk (n = 100)			
1-5	15 (15.0)	9 (18)	6 (12)
6-10	34 (34.0)	14 (29)	20 (39)
11-20	12 (12.0)	6 (12)	6 (12)
>20	25 (25.0)	16 (33)	9 (18)
Not sure	14 (14.0)	4 (8)	10 (20)
HF training			
None	46 (45.1)	24 (48)	22 (42)
A little	31 (30.4)	13 (26)	18 (35)
Some	23 (22.5)	13 (26)	10 (19)
A lot	2 (2.0)	0	2 (4)
Management (CC-SCHFI), median (IQR)^b^	60.0 (40.0-70.0)	60.0 (45.0-70.0)	55.0 (35.0-65.0)
Maintenance (CC-SCHFI), median (IQR) (n = 101)^b^	86.7 (76.7-93.3)	86.7 (76.7-93.3)	86.7 (76.7-93.3)

Abbreviation: CC-SCHFI, Caregiver Contribution to Self-Care in HF Index; EUC, enhanced usual care; HF, heart failure.

^a^ Other race included the following responses: Black/Puerto Rican, Latino, Hispanic, Caribbean, Dominican Republic, and Mestizo.

^b^ The CC-SCHFI is split into 3 subdomains: HF maintenance, HF management, and HF caregiving self-efficacy (confidence), each scored independently (range, 0-100; higher score indicates greater maintenance or management).^23^

With respect to experience with HF, most HHAs (65 HHAs [63.7%]) had cared for 5 or fewer patients with HF in the past (Table 1). With respect to time spent with patients with HF, 15 (15.0%) spent 0 to 5 hours per week; 34 HHAs (34.0%), 6 to 10 hours per week; 12 HHAs (12.0%), 11 to 20 hours per week, and 25 HHAs (25.0%), 20 or more hours per week. With respect to prior HF training, 46 HHAs (45.1%) reported they had none, 31 HHAs (30.4%), a little; 23 HHAs (22.5%), some; and 2 HHAs (2.0%), a lot. They had a high median (IQR) contribution to HF maintenance care score, at 86.7 (76.7-93.3) points.

At baseline, participants had an overall median (IQR) DHFKS score of 7.0 (5.0-8.0) points and a median (IQR) self-efficacy score of 75.0 (62.5-82.5) points. (Table 2). At baseline, 58 HHAs (56.9%) reported 911 calls that could have been prevented if they had been able to reach the nurse, and 46 HHAs (45.1%) reported 911 calls that could have been prevented if they had been able to reach the physician. After randomization, there were no significant differences in sociodemographics, employment, HF experience, or outcomes at baseline. (Table 1 and Table 2)

**Table 2.** Baseline Measures for Primary and Secondary Outcomes

Outcomes	Total (N = 102)	EUC (n = 50)	Intervention (n = 52)	*P* value
Co–primary outcomes				
DHFKS, median (IQR)	7.0 (5.0-8.0)	7.0 (6.0-8.0)	6.0 (5.0-8.0)	.10
Self-efficacy, median (IQR)	75.0 (62.5-82.5)	72.5 (62.5-77.5)	76.2 (62.5-82.5)	.19
Secondary outcomes				
Agreed with statement “I have called 911 when that could have been prevented if I had been able to reach my supervisor/the nurse.” No. (%)	58 (56.9)	30 (60.0)	28 (53.8)	.52
Agreed with statement “I called 911 when that could have been prevented if supervisor and I had been able to reach the clients’ doctor.” No. (%)	46 (45.1)	20 (40.0)	26 (50.0)	.35

Abbreviations: DHFKS, Dutch HF Knowledge Scale; EUC, enhanced usual care.

^a^ Range, 0-15; higher scores indicate greater knowledge.

^b^ Range, 0-100; higher scores indicates greater self-efficacy.

^c^ Defined by the question: “I have called 911 when that could have been prevented if my supervisor and I had been able to reach the client’s doctor.” Responses, on a 7-point Likert scale, were dichotomized into agree, strongly agree, and very strongly agree vs all other responses.

^d^ Defined by the question: “I have called 911 when that could have been prevented if I had been able to reach my supervisor/the nurse.” Responses, on a 7-point Likert scale, were dichotomized into agree, strongly agree, and very strongly agree vs all other responses.

### Change in HF Knowledge and HF Caregiving Self-Efficacy

**Figure 2.**
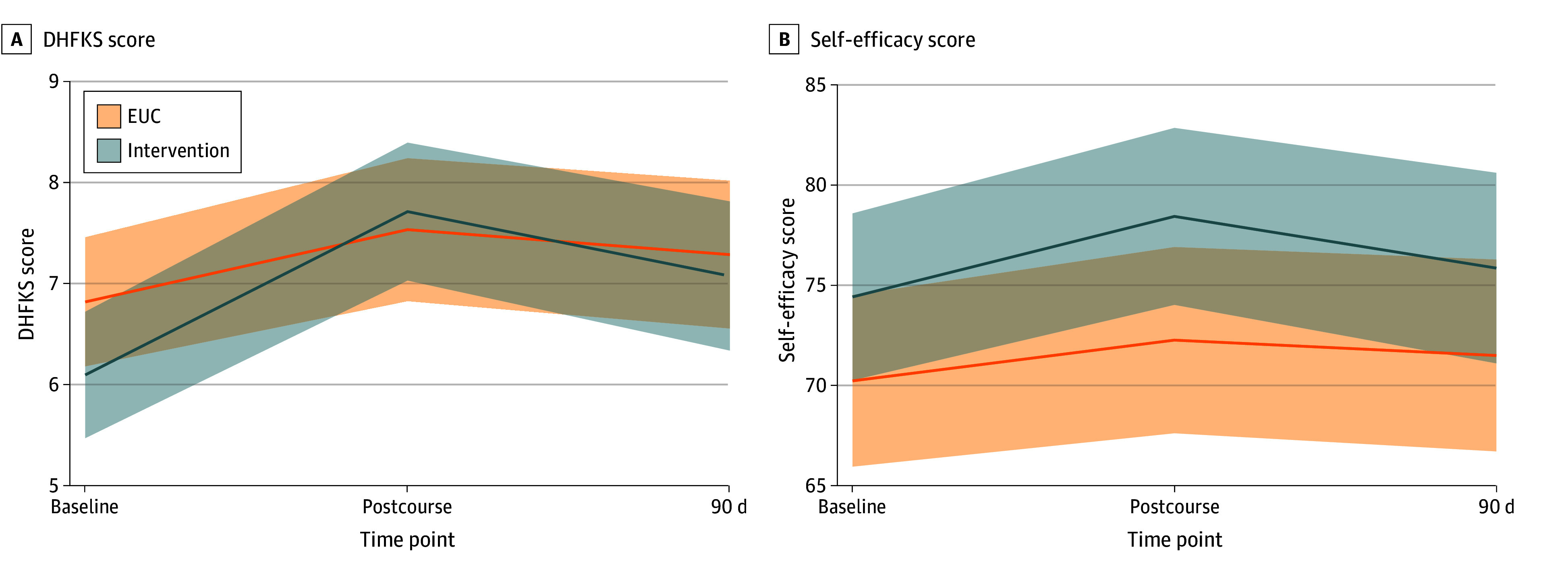
Model-Based Outcome Trajectory by Study Group Mixed linear regression model estimated outcome with 95% CI at baseline, postcourse and 90-day assessments. A, DHFKS indicates Dutch Heart Failure Knowledge Scale (range, 0-15; higher scores indicate greater knowledge. B, Self-efficacy scores range from 0 to 100; higher scores indicate greater self-efficacy. Intervention vs enhanced usual care (EUC): postcourse difference, 1.99 (95% CI, −4.71 to 8.68); *P* = .56; 90-day difference, 0.17 (95% CI, −6.83 to 7.17); *P* = .96.

The HF training course was delivered to 83 participants (81.4%). including 40 in the EUC group and 43 in the intervention group, with the rest being lost to follow-up (Figure 1). All HHAs who engaged in the course completed the full training. DHFKS scores were significantly higher after the course compared with baseline (difference, 1.62 [95% CI, 0.83 to 2.41] points; *P* = .001) and at 90 days (difference, 0.98 [95% CI, 0.83 to 2.41] points; *P* = .02) compared with baseline among participants who received the intervention (Figure 2). However, there were no significant differences in the change of DHFKS scores between participants in the intervention group vs the EUC group at postcourse or 90-day assessments. There were no significant differences in DHFKS scores between participants in the intervention group vs EUC group after the course (difference, 0.90 [95% CI, −0.23 to 2.04] points; *P* = .12) or at the 90-day assessment (difference, 0.51 [95% CI −0.67 to 1.69] points; *P* = .40).

Self-efficacy scores were not significantly different between time points among those in the EUC vs the intervention group. Additionally, self-efficacy scores were not significantly higher at course completion or 90 days when comparing participants in intervention vs EUC (postcourse: difference, 1.99 [95% CI, −4.71 to 8.68] points; *P* = .56; 90 days: difference, 0.17 [95% CI, −6.83 to 7.17] points; *P* = .96).

Participants with the lowest DHFKS scores at baseline had the largest gains. Among participants who received the intervention, DHFKS scores increased significantly between baseline and postcourse assessments (difference, 2.57 [95% CI, 1.78 to 3.36] points; *P* < .001) and between baseline and 90 days (difference, 1.88 [95% CI, 1.05 to 2.71] points; *P* < .001) (eFigure 1 and eTable 1 in Supplement 2). This was seen in the EUC group as well: DHFKS scores increased significantly between baseline and postcourse assessments (difference, 1.88 [95% CI, 1.03 to 2.72] points; *P* < .001) and baseline and 90-day assessments (difference, 0.93 [95% CI, 0.05 to 1.81] points; *P* = .04). However, the difference in change in scores between EUC and intervention groups was not significant.

This pattern, where participants with the lowest scores at baseline had the greatest gains, was also seen for self-efficacy. Among participants who received the intervention, SE scores increased significantly between baseline and postcourse assessments (difference, 9.23 [95% CI, 3.78 to 14.68] points; *P* < .001) and baseline and 90-day assessments (difference, 8.28 [95% CI, 2.56 to 14.00] points; *P* = .005) (eFigure 1 and eTable 2 in Supplement 2). Among participants who received EUC, self-efficacy scores increased significantly between baseline and postcourse assessments (difference, 6.27 [95% CI, 1.54 to 11.00] points; *P* = .009) and between baseline and 90-day assessments (difference, 7.91 [95% CI, 3.18 to 12.64] points; *P* = .001). However, the difference in change in score between EUC and intervention groups was not significant.

**Figure 3.**
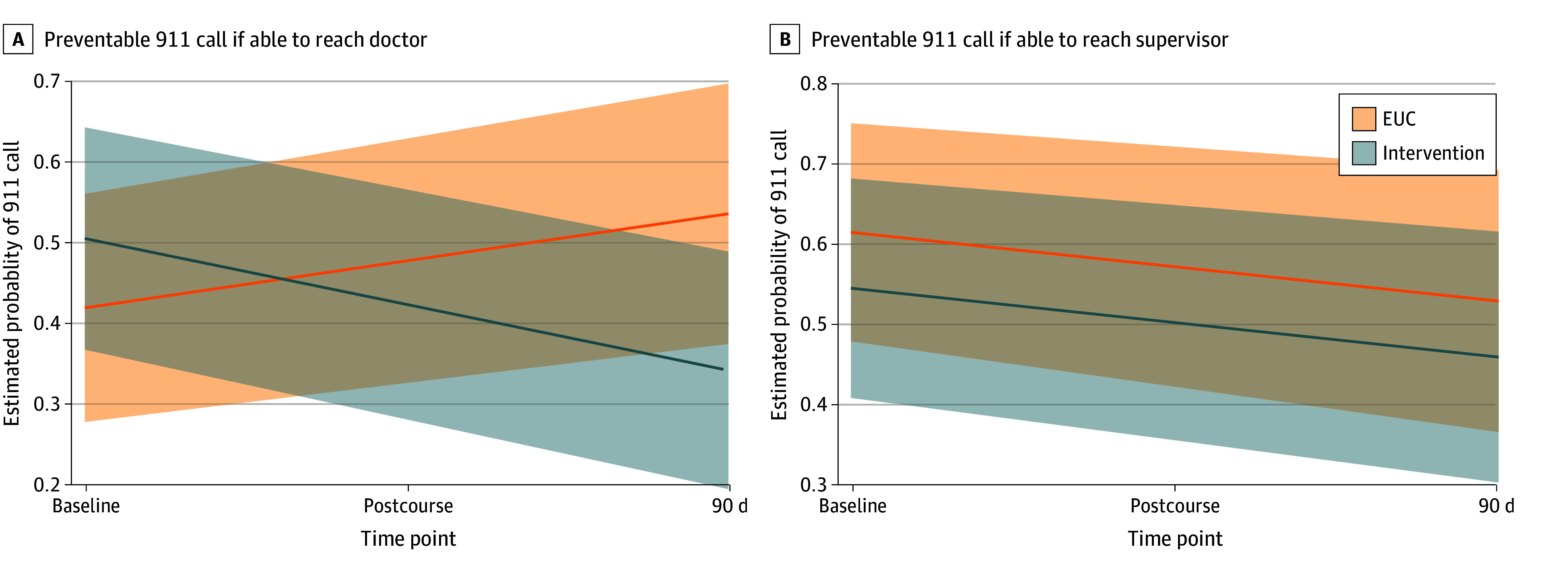
Mixed Logistic Regression Model Estimated Outcome at Baseline, Postcourse, and 90-Day Assessments Preventable 911 calls if able to reach doctor were defined by the question: “I have called 911 when that could have been prevented if my supervisor and I had been able to reach the client's doctor.” Preventable 911 calls if able to reach supervisor were defined by question: “I have called 911 when that could have been prevented if I had been able to reach my supervisor/the nurse.” Responses for both questions included a 7-point Likert scale, and both questions were dichotomized into agree, strongly agree, and very strongly agree vs all other responses.

At 90 days, there was no statistically significant within-group differences in the proportion of HHAs reporting preventable 911 calls (intervention: 0.51 [95% CI, 0.37-0.64] at baseline vs. 0.34 [95% CI, 0.2-0.49] at 90 days; *P* = .06; EUC: 0.42 [95% CI, 0.28-0.56] at baseline vs 0.54 [95% CI, 0.38-0.70] at 90 days; *P* = .21). The difference between groups was statistically significant (*P* = .04) (Figure 3).

### Exploratory Patient Outcomes

In this cohort of 102 HHAs, there were 82 unique HHA-patient with HF pairs (eFigure 2 in Supplement 1). After exclusions, we had an analytic sample of 58 patients. Over the course of 90 days, there were a total of 24 ED visits, with 15 among patients cared for by HHAs in the EUC group and 9 among patients cared for by HHAs in the intervention group. This study was not statistically powered for assessing this outcome, so our finding of an incidence rate ratio of 0.56 (95% CI, 0.25 to 1.28) (*P* = .17) for ED visits within 90 days among patients cared for by intervention HHAs compared with patients cared for by EUC HHAs should be considered exploratory.

Overall, there were 6 hospitalizations among the patients in the cohort, 2 among patients cared for by HHAs in the EUC group, and 4 among patients cared for by HHAs in the intervention group. Due to small numbers of hospitalizations, we were unable to model this outcome.

### Engagement With Intervention Among HHAs

Of 40 HHAs who were eligible to message the nurse (ie, received initial message from nurse to begin the chat), 26 (65.0%) engaged and 14 (35.0%) did not. Of 26 HHAs who engaged, 11 (42.3%) conversed regularly and were considered high-level users.

Messages had 5 main types, from most common to least common: (1) HHA observes and conveys clinical status of patient to nurse (eg, “her shortness of breath improved”); (2) HHA collects and transmits vital signs to nurse (eg, “blood pressure is 167/71 heart 64 sugar is 150”), (3) HHAs and nurses discuss health care utilization (eg, “I called the PCP’s [primary care physician’s] office to secure an early appointment.”), (4) HHA counsels patient (eg, “elevate legs”), (5) nurse advised HHA (eg, “consistent weight [for your patient] is a good sign”). Additional data on engagement, feasibility, and acceptability of the intervention will be reported elsewhere.

## Discussion

In this pilot RCT among HHAs caring for patients with HF, we found that HF training improved HF knowledge and caregiving SE among all HHAs. The largest gains for both outcomes were among HHAs with the lowest HF knowledge and self-efficacy at baseline, a finding that was both statistically and clinically significant.^10,16,23,24,25^ While the chat feature (intervention) did not significantly improve primary outcomes, it significantly reduced self-reported preventable 911 calls among HHAs in the intervention group, a secondary outcome. While our study was not statistically powered for assessing patient-level outcomes, our finding regarding incidence of ED visits among patients cared for by intervention HHAs warrants further investigation. Overall, our findings suggest that the intervention has potential to reduce excess health care utilization among HF patients receiving home care.

To our knowledge, this is the first trial to focus on HHAs as a workforce that can be empowered and integrated to improve care for patients with HF, a rapidly growing, high-need population with high risk for morbidity, hospitalization, and mortality.^1,2^ While prior research has identified HHAs as a potential group of health care practitioners, often observing and advising patients in the home, they have rarely been the focus of interventions aimed at improving health care delivery in HF. Rather, interventions that engage caregivers have focused on family members, and those that engage home-based clinical practitioners have focused on clinicians (eg, nurses, physical therapists), pharmacists, or community health workers.^26,27,28,29,30,31^ While some of these have been successful, most have not been sustainable or affordable in the US health care system.^32,33,34^ Our findings advance the notion that HHAs, who are already funded by Medicaid and Medicare (albeit sometimes privately), are frontline health care practitioners with untapped potential to enhance and improve care in HF. Our prior work has found that HHAs frequently care for adults with HF and that training HHAs in key aspects of HF care was associated with improved knowledge and confidence.^16^ However, to our knowledge, this had not been previously evaluated in the context of a trial, nor among HHAs caring for patients with HF in real time. In this study, we advance the science by addressing both training and communication challenges that this workforce faces. Not only did we find that HF training improved all participants knowledge and confidence, but many HHAs who were given the opportunity to communicate more rapidly with their clinical supervisors did so. When they did, it was most often about clinical care. Doing so was associated with fewer preventable 911 calls among HHAs in the intervention group. Notably, HHAs in the EUC group had higher rates of preventable 911 calls at 90 days. It is plausible that training HHAs on HF may have primed them toward recognizing and responding to HF symptoms, but without easier ways to reach team members. These findings suggest that HHAs are indeed a workforce that, when trained and properly integrated into the care team, can contribute to better care in the home and reduce avoidable trips to the ED, a finding that warrants testing in a larger full-powered trial.

The chat feature intervention tested in this trial represents an important first step toward offering HHAs real-time technology solutions. A prior scoping review found that although numerous mHealth applications exist for HHAs, most focus on documentation of care tasks (ie, completion of tasks), timesheets and clocking in and out of work, and shift booking.^35^ Indeed, very few mHealth apps are designed to support the actual care that HHAs provide to patients in the home. Although not all HHAs engaged in the chat, when app messages were sent, most HHAs reported their clinical observations (signs, symptoms, vital signs) to nurses, discussed calling physicians, and received guidance. While we were able to calculate the frequency of the types of message HHAs sent to their nurses, we were unable to examine the frequency and type of messages (eg, transmitting vital signs) and patient outcomes, but we aim to do so in future studies.

It is worth noting that despite a lack of prior HF training and low levels of HF knowledge, participants in the trial reported high levels of confidence with HF caregiving at baseline. While this phenomenon, known as the Dunning-Kruger effect (a cognitive bias in which people overestimate ability),^36^ is common among numerous health care workers, there may be other more dominant reasons why we are seeing this among HHAs in our trial. First, many of the trial participants had been HHAs for a long time, with most working more than 5 years and 25% working more than 15 years. Second, most participants had worked with patients with HF in the past, with 17% working with more than 5. This signals that HHAs in our study may indeed have felt highly comfortable caring for patients with HF in the home, many of whom were older adults with multiple chronic conditions, even if actual concrete HF knowledge was low. Notably, like other studies with HHAs, we found that those with the lowest levels of knowledge and confidence at baseline saw the greatest gains in both knowledge and confidence after training. This alongside the finding that 42% of HHAs in the intervention group were high-level users of the chat feature suggest that interventions may not be a 1-size-fits-all solution for this workforce but may be most effective when tailored to certain HHAs (ie, low knowledge, low confidence) and their patients (high clinical needs in which communication is warranted).

### Strengths and Limitations

Strengths of the study include its randomized controlled design with HHAs caring for adults with HF and that it was conducted in partnership with one of the largest LHCSAs in New York, New York, which serves a high number of patients with HF across downstate New York. Additionally, the co–primary outcomes for HHAs used validated survey scales. Retention rates were high, considering and despite HHAs’ challenging employment structure, often characterized by high caseloads, long commutes, lengthy shifts, and low wages that leave them with little free time for personal or professional engagements beyond their work.^7^ Furthermore, the interventions were previously developed with HHAs and home care leaders themselves and principles of user-centered design.^20^

However, we also note several limitations. First, although HHAs were diverse in language, race, ethnicity, experience, and borough in which they provided care, the study was conducted at a single large LHCSA in New York, New York. Most home care organizations are smaller and serve fewer patients and may not be as amenable to technological innovation, which may limit generalizability of our findings. Furthermore, while feasibility was demonstrated, engagement with the mHealth app was not as high as it could have been, which has the potential to bias the results toward the null. Second, our HHA-level outcomes were self-reported, which may introduce recall bias. Third, participants and research staff were not blinded with respect to intervention delivery.^37,38,39^ Fourth, although we were able to ascertain patient outcomes, we were unable to characterize their HF severity or other clinical comorbidities, which may have contributed to ED visits or hospitalizations. Additionally, our exploratory analysis of participants with low baseline DHFKS and self-efficacy scores may have the risk of regression to the mean.

## Conclusions

In this pilot RCT of HHAs caring for patients with HF, we found that HF training improved HHAs’ HF knowledge and HF caregiving self-efficacy, with the largest gains among those with the lowest scores at baseline. While the ability to message nurses did not improve primary outcomes, it reduced self-reported preventable 911 calls compared with the EUC group. Our finding regarding incidence of ED visits among patients cared for by intervention HHAs warrants further investigation, as our study was not powered to assess patient-level outcomes. The findings of this study can inform the design of a future large-scale trial to better support and integrate HHAs providing care HF care. Interventions and studies like these are greatly needed to support this essential workforce and a rapidly aging population in which HF is increasingly prevalent.

## Notes

Supplement 1.
Trial Protocol and Statistical Analysis Plan

Supplement 2. **eFigure 1.** Model based Outcome Trajectory by Arm Among Participants with Low Baseline score **eFigure 2.** Exclusion cascade for the analysis of patient outcomes **eTable 1.** Participant (Home health aide) characteristics by baseline DHFKS **eTable 2.** Participant (Home health aide) characteristics by baseline Self-efficacy

Supplement 3.
Data Sharing Statement
