# Supplementary material for: Home Health Aides Caring for Adults With Heart Failure: A Pilot Randomized Clinical Trial
Source: JAMA Netw Open. 2025 Nov 10;8(11):e2548121. doi: 10.1001/jamanetworkopen.2025.48121 (PMC12603854; doi:10.1001/jamanetworkopen.2025.48121)
Supplement: Supplement 1. — Trial Protocol and Statistical Analysis Plan [file jamanetwopen-e2548121-s001.pdf]

Author Manuscript
Author Manuscript
Author Manuscript
Author Manuscript

HHS Public Access
Author manuscript
Contemp Clin Trials. Author manuscript; available in PMC 2024 August 01.

Published in final edited form as:
Contemp Clin Trials. 2024 August ; 143: 107570. doi:10.1016/j.cct.2024.107570.

Leveraging Home Health Aides to Improve Outcomes in Heart Failure: A Pilot Study Protocol

Cisco G. Espinosa, BA[1], **Sasha Vergez, BA, BS**[2], **Margaret V. McDonald, MSW**[2], **Monika Safford, MD**[1], **Jacklyn Cho, BS**[3], **Jonathan N. Tobin, PhD**[4], **Omar Mourad, BS**[5], **Rosa Marcus**[2], **Joanna Bryan, MPH**[1], **Samprit Banerjee, PhD**[1], **Nicola Dell, PhD**[6], **Penny Feldman, PhD**[2], **Madeline R. Sterling, MD, MPH, MS**[1]

[1.]Weill Cornell Medicine, New York, NY

[2.]Center for Home Care Policy & Research, VNS Health, New York, NY

[3.]Renaissance School of Medicine at Stony Brook University, Stony Brook, New York, USA.

[4.]Clinical Directors Network (CDN) and The Rockefeller University Center for Clinical and Translational Science, New York, NY

[5.]Weill Cornell Medicine- Qatar, Qatar Foundation - Education City, Doha, Qatar

[6.]Cornell Tech, New York, NY

Abstract

Heart failure (HF) affects six million people in the U.S., is associated with high morbidity, mortality, and healthcare utilization.[1,2] Despite a decade of innovation, the majority of interventions aimed at reducing hospitalization and readmissions in HF have not been successful.[3-7] One reason may be that most have overlooked the role of home health aides and attendants (HHAs), who are often highly involved in HF care.[8-13] Despite their contributions, studies have found that HHAs lack specific HF training and have difficulty reaching their nursing supervisors when they need urgent help with their patients. Here we describe the protocol for a pilot randomized control trial (pRCT) assessing a novel stakeholder-engaged intervention that provides HHAs with a) HF training (enhanced usual care arm) and b) HF training plus a mobile health application that allows them to chat with a nurse in real-time (intervention arm). In collaboration with the VNS Health of New York, NY, we will conduct a single-site parallel arm pRCT with 104 participants (HHAs) to evaluate the feasibility, acceptability, and effectiveness (primary outcomes: HF knowledge; HF caregiving self-efficacy) of the intervention among HHAs caring for HF patients. We hypothesize that educating and better integrating HHAs into the care team can improve their ability to provide support for patients and outcomes for HF patients as well (exploratory outcomes include hospitalization, emergency department visits, and readmission). This study offers a novel and potentially scalable way to leverage the HHA workforce and improve the outcomes of the patients for whom they care.

Clinical trial.gov registration: NCT04239911

Disclosures
Dr. Safford is the founder of MedExplain, Inc., an independent company focusing on integrating patient-centered health information into the clinical care process. The other co-authors declare that there are no conflicts of interest.

Author Manuscript Author Manuscript Author Manuscript Author Manuscript

Keywords

home health; home health aides; heart failure; long term care; clinical trial design

1. Introduction

Heart failure (HF) affects 6.2 million people in the U.S, is associated with a high risk of disability, morbidity and mortality, and is responsible for 1 million hospitalizations per year.[1,2] Over 20% of Medicare beneficiaries hospitalized for HF are readmitted within 30 days and up to 50% within 6 months.[3,4,14] Hospitalizations and readmissions contribute to poor health and are costly for the healthcare system.[3,15]

Although numerous interventions have tried to reduce hospitalizations and readmissions in HF, few have had sustained success.[5,6,16] One reason may be that most have not considered the feedback of healthcare professionals involved in HF care, including in the home.[8-13] Nearly a third of HF patients receive home-based care from certified and licensed home care agencies.[17] Included in this care are home health aides and attendants (HHAs), a fast-growing healthcare workforce, who provide assistance with personal care and health-related tasks.[13,18-22] Prior studies have found that HHAs are involved in key aspects of HF care, including monitoring for signs and symptoms of the disease, preparing meals, and reminding patients to take medication.[23, 24]

Despite this involvement, prior studies have found that 65% of HHAs lack HF training and 60% lack confidence in providing HF care.[23, 25-28] Compounding this, HHAs report difficulty reaching their supervisors at their home care agency (e.g. a nurse or a clinical coordinator) by telephone when they have questions or need clinical support.[23, 29, 30] This can result in HHAs calling 911 and subsequent emergency department (ED) visits (Appendix A). Indeed, in a recent survey of 391 HHAs, nearly half (48%) of HHAs self-reported that their 911 calls could have been prevented if they had been able to reach their nurse supervisor or their patients' doctor.[31]

To address these gaps, we previously developed a HF training course for HHAs and refined an existing mobile health (mHealth) application (app) for HHAs which allows them to text "chat" their nurse in real-time.[32,33] The goal of this pilot randomized control trial (pRCT) is to examine the feasibility, acceptability, and effectiveness of these interventions among agency-employed HHAs caring for community-dwelling adults with HF. This paper describes the research protocol for this pRCT.

2. Methods

2.1 Study Objectives

This study aims to improve HHAs' ability to care for HF patients through intervention(s) that increase their HF knowledge and HF caregiving self-efficacy. The long-term goal is to improve outcomes of HF patients by training and integrating HHAs into the care team, a concept we will formally evaluate in the study that follows. The two main objectives of this pRCT are to test the : 1) feasibility and acceptability of an education and communication-

Author Manuscript Author Manuscript Author Manuscript Author Manuscript

based intervention (HF training course and mHealth app chat tool) among HHAs caring for adults with HF receiving home care; 2) effectiveness of the intervention on HHAs' HF knowledge and caregiving self-efficacy, compared to HHAs who receive the control condition (HF training alone); 3) effect of the intervention on HF patients' outcomes, including hospitalizations, ED visits, readmissions, and quality of life, in an exploratory fashion. We hypothesize that: (H1): the intervention will be feasible (> than 80% of HHAs enrolled will complete the study) and acceptable (> 90% of HHAs enrolled will find it acceptable); (H2): there will be statistically significant differences with respect to the primary outcomes (HF knowledge and caregiver self-efficacy) between the study groups (enhanced usual care vs. intervention). Although not powered to detect differences in patient outcomes, we hypothesize that patients with HHAs in the intervention group will have fewer adverse outcomes than those in the control group.

2.2 Study Setting

The study involves a collaboration between an academic medical center, Weill Cornell Medicine, and VNS Health, a large, non-profit organization that provides home care, population and care management, community health services, and hospice care, both of which are in New York, NY. VNS Health also includes the Center for Home Care Policy & Research. Within VNS Health, this study will be conducted with the Licensed Certified Home Service Agency (LCHSA), Personal Care, which employs over 6,500 HHAs.[34] HHAs at Personal Care are licensed and certified to provide personal care, and typically serve one patient for an extended period. In addition to initial training and certification requirements, HHAs receive 12 hours of annual in-service education, although to date, they have not received dedicated HF training. All HHAs at Personal Care are expected to use an mHealth app, "CareConnect," a human capital management technology company which assists LCHSAs with managing and scheduling. CareConnect also provides eLearning content to HHAs and secure communications for field-based caregivers (HHAs and nurses).[35] Prior to this study, the "chat" feature had not been used among HHAs.

2.3 Study Design

This is a single-site parallel arm pRCT, that plans to enroll 104 participants (HHAs) and randomize them in a 1:1 ratio into the enhanced usual care or intervention arm. Participants are in the trial for 90 days each within their respective arms (Figure 1). Participants in both arms receive the HF training course, whereas only those in the intervention arm receive the mHealth app chat tool (sections 2.10, 2.11). Data are collected from participants on days 0, 45, and 90 (section 2.11). Recruitment and follow-up are ongoing through the end of 2023.

2.4 Stakeholder Engagement

All aspects of the study and this protocol (e.g., design, procedures, etc.,) were developed with stakeholder engagement, including experts in home care, HHAs, agency staff, clinicians (nurses, primary care, geriatrics, and cardiology), family caregivers, and patients. For example, the rationale for conducting this pRCT was informed by focus groups, interviews, and surveys with stakeholders [23,26,28,36,37] and the interventions were developed and refined in partnership with HHAs and nurses[29,30,33,38]. To develop this protocol, the

Author Manuscript

research team met weekly with key leaders and staff at VNS Health and Partners in Care over one year.

2.5 Study sponsorship and Institutional Review Board Approval

This study is supported by the National Heart, Lung, and Blood Institute of the NIH (K23HL150160). The content is solely the responsibility of the authors and does not necessarily represent the official views of the NIH. This trial is registered (ClinicalTrials.gov: NCT04239911) and approved by the Institutional Review Board of Weill Cornell Medicine (IRB # 19-08020553) and VNS Health (IRB # E19-003).

2.6 Eligibility Criteria

To be eligible, HHAs will need to: speak English or Spanish, be employed by Personal Care of VNS Health, provide care (during the study) to a HF patient considered vulnerable to unplanned emergent care events (as defined by VNS Health) and be enrolled in a managed long-term health care plan. HF was defined using ICD-10 codes of I09.9, I11.0, I13.0, I13.2, I25.5, I42.0, I42.5 - I42.9, I43.x, I50.x, P29.0. To be eligible, HHAs will also need to have a smartphone and/or an email address. To recruit HHAs caring for HF patients, this study requires access to patient records, granted through a HIPAA waiver of authorization; the intervention will not be conducted with patients.

2.7 Recruitment of HHAs

A research assistant (RA) will identify HHAs caring for HF patients and assess their interest and eligibility by telephone. For those who are not interested, the RA will document their reason(s) for declining. Participants who are eligible and interested will be scheduled for an in-person onboarding session at VNS Health. At this session, the RA will provide an overview of the study. Participants will provide written (electronic) informed consent, complete a baseline survey (section 2.11, Appendix B), and their Smartphone will be registered. They will then be randomized to a study arm using a block randomization design (blocks of 4 and 6 using the R package 'blockrand'). Those assigned to the intervention arm will be oriented to the mHealth app chat tool with another RA. After onboarding, participants will receive a $25 gift card (section 2.11). Regardless of study arm, all participants will be scheduled to attend the virtual HF training course (section 2.9).

2.8 Recruitment of nurses

Four nurses from VNS Health will be recruited to the intervention arm. To be eligible, nurses will need to be office-based and provide clinical oversight of HF patients. Their role will be to respond to chat messages from HHAs in the intervention arm regarding their HF patients (section 2.11). After providing verbal consent, nurses will be oriented to the study and the mHealth app messaging feature. Nurses will receive $100 compensation.

2.9 Enhanced Usual Care Arm (HF Training Course and E-Learning Resources)

In line with our prior stakeholder-engaged research[32,36,39]and feedback from VNS Health, all participants will receive the (virtual) HF training course. The development and piloting of the course has been previously described.[33,38] Briefly, the 1.5-hour course was developed to

Author Manuscript Author Manuscript Author Manuscript Author Manuscript

meet HHAs' educational needs while aligning with their scope of care. The course, delivered via Zoom, provides HHAs with an overview of HF, including its signs and symptoms, with most of the education occurring through facilitated case-based learning. Cases review symptom recognition and monitoring, lifestyle behaviors, medication adherence, and triaging emergencies in the home. Finally, HHAs learn how to communicate with patients, family members, and other healthcare providers.[32,36,37] The course is delivered and moderated by a trained instructor (including clinicians, RAs, agency staff).

In line with HHAs' desire to access course content "on-demand," we created a website so they can access content for free and at any time. For example, within the website they can access a symptom triage tool (https://cornellhomecare.com/trafficlight) or read about key topics through short, evidence-based articles on the Patient Activated Learning System, for example "What is Heart Failure (https://cornellhomecare.com/basics/heart-failure).[39]

2.10 Intervention Arm (mHealth App Chat Tool)

Participants in the intervention arm will receive both the HF training course and the mHealth app chat tool (via CareConnect). The chat tool enables HHAs to text the study nurse in a HIPAA compliant manner when caring for their HF patient. Additionally, the app links the HHA and nurse in a chat thread which can be revisited by the HHA or nurse during the study period. In preparation for this pRCT, we refined the chat tool with HHAs, nurses, VNS Health and CareConnect staff and created orientation videos and materials for study participants. Participants will be instructed to chat with the nurse when they have questions regarding their HF patient (i.e., symptoms arise), b) they need help with the patients' care; c) they want to provide an update. Non-clinical questions will be directed to typical care pathways (e.g., calling their coordinator).

At the start of the study period (after randomization and orientation), the nurse assigned to the participant (HHA) will initiate a "test" chat to ensure a working connection in CareConnect. After this is received and answered, participants can message their nurse. Automatic SMS text -based reminders (outside of CareConnect) will be sent through Twilio to reinforce the behavior change of HHAs chatting directly with the nurse during the beginning of the study period.

2.11. Data Collection

2.11.A Study Timeline—Each participant will be involved for 90 days after completing the HF training course. There are four data collection points for each HHA participant (Table 1).

2.11.B Data Collection and Data Sources—We will collect data using a multi-modal approach (Table 1). This will include surveys, observation, usage data (i.e., course website, chat feature), interviews, and claims-based data. Surveys will be administered through Research Electronic Data Capture (REDCap) software; a secure data management system.

Participants will complete a baseline survey (T=0) that includes sociodemographic and employment data and baseline HF knowledge and HF care-giving self-efficacy (Appendix B). After taking the HF training course, participants will complete a brief survey to assess

Author Manuscript Author Manuscript Author Manuscript Author Manuscript

knowledge and attitudes towards the course (T=1). Throughout the study period, use of the course website will be monitored by the research team with Google analytics. The chats will be monitored weekly for quality control purposes and content will be analyzed formally at the end of the trial (section 2.13.A). At 45 days (T=2) participants will be interviewed about their experiences in the study as well as their use of study materials and the chat (intervention only). At 90 days (T=3), participants will complete a survey which assesses HF knowledge, HF caregiving self-efficacy, attitudes towards their job, communication practices, and their patients' status. A subset of participants in both arms will be interviewed about their experiences in the study. At 90 days (T=3) we will also interview a sub-set of patients to assess their experiences receiving home care, perspectives on the role of HHAs, and their overall health, including their quality of life. Patient outcomes (hospitalization, readmissions, etc.) will be assessed by health services use claims.

All participants receive a $25 gift card for each completed data collection point.

2.12 Study Outcomes

The three main outcomes are feasibility, acceptability, and effectiveness.

2.12.A Feasibility—To asses feasibility, the RE-AIM implementation framework will be used, which stands for: Reach, Efficacy/Effectiveness, Adoption, Implementation, and Maintenance.[40-42] The framework was modified in 2019 to include organizational and policy lenses; this version, the RE-AIM/PRISM, is well-suited to this pRCT.

Reach will be assessed by tracking the number of participants (HHAs) approached and enrolled, and reasons why they declined; Effectiveness through baseline and follow-up assessments; Adoption by the number who completed the study orientation (both arms), HF training course (both arms), chat tool orientation (intervention arm), and completed the trial (both arms). With respect to implementation, data will be collected on fidelity to the study protocol (defined as the extent to which the intervention was delivered as planned [section 2.12.B]), intervention adaptations, completion rate of intervention components, and staff required to deliver the intervention, among others (Table 2). Maintenance will be assessed in a future study.

2.12.B Fidelity—A fidelity checklist was established to record the quality and consistency of participant and study staff activities at various points (Appendix C). The checklist ensures that RAs, nurses, and course instructors all have appropriate training and orientation to their roles (responding to chats, recruiting, and onboarding, teaching the training course, etc.). The checklist places emphasis on maintaining a consistent recruitment process. It also ensures that all participants receive the intervention as intended.

2.12.C Acceptability—Acceptability will be assessed with a brief survey after the training course and through qualitative interviews with HHAs, nurses, and staff (Table 1 [section 2.11, Appendix D]). Semi-structured interviews will be conducted by RAs. Participants from both arms will be interviewed about their experiences with the course; those in the intervention arm will be asked about their experience with the chat feature. Nurses will be asked about their experiences receiving and responding to messages

Author Manuscript Author Manuscript Author Manuscript Author Manuscript

from HHAs, the interventions' impact on workflow, and their overall experience in the trial. Additionally, after receiving verbal consent, 30 patients (intervention arm) will be interviewed to understand their experiences.

2.12.D Effectiveness—Effectiveness will be assessed with two primary outcomes: 1) HF knowledge, using the Dutch HF Knowledge Scale (DHFKS)[43] and 2) HF caregiving self-efficacy, assessed within the Caregiver Contribution to Self-Care in HF Index (CC-SCHFI).[44,45] The DHFKS is a 15-item scale which measures HF knowledge including treatments and symptom recognition. Scores range from 0-15, with higher scores indicating higher knowledge. The CC-SCHFI is a 22-item scale which measures caregivers' contributions to: HF maintenance, HF management, and HF caregiving self-efficacy (confidence). Each sub-scale is scored independently. The third sub-scale, also known as the 10-item HF caregiving self-efficacy scale, will be the main outcome. HF caregiving self-efficacy scores range from 0-100, with higher scores indicating greater self-efficacy. This scale has previously been validated (Cronbach $\alpha = 0.94$)[44] by our team among HHAs (Factor Score Determinacy Coefficient 0.80 – 0.87).[26]

Two secondary outcomes include job satisfaction, assessed with the Work Domain Satisfaction Scale,[47] and intention to leave, assessed with the Turnover Intention scale[48] (Appendix B). The WDSS is a 5-item scale which measures job satisfaction; scores range from 5-20, with higher scores indicating greater satisfaction. The Turnover Intention scale is a 2-item scale which measures workers' intentions of leaving their current job and searching for a new job (in another field) in the next year (Appendix B).

Patient outcomes include all-cause ED visits, hospitalization, readmission and quality of life. These outcomes will be ascertained from electronic health records data (dates and reason for service use disruption) and telephone-based surveys. Quality of life will be assessed with the Kansas City Cardiomyopathy Questionnaire (KCCQ-12), a 12-item validated scale that evaluates patient's perceptions of their health status with particular focus on HF symptoms within a 2-week recall period.[48]

2.13 Statistical Methodology

2.13.A Analytical Plan for Quantitative Data—Data from REDCap will be exported to STATA for analysis. Descriptive statistics will be done to characterize the sample. Differences in participant characteristics by study arm will be examined using tests of association (chi-squared and t-tests for normally distributed data). We will also compare the characteristics of participants who consented and enrolled to those who declined to participate.

To determine the effect of the intervention on the primary outcomes, we will compare HF knowledge and HF caregiving self-efficacy scores by trial arm using paired t-tests for normally distributed data or Wilcoxon signed rank test for non-parametric data. We will examine the effect of randomization on the change in scores using linear regression models. Primary analyses will be conducted using intention-to-treat, when appropriate. We will examine participant characteristics across trial arms and adjust for covariates that are imbalanced ($p < 0.10$). We will conduct the same analyses for the secondary outcomes.

Author Manuscript Author Manuscript Author Manuscript Author Manuscript

We will perform exploratory analyses to examine the impact of the intervention on patient outcomes; to do so, we will use hierarchical logistic mixed effects regression models to account for clustering of patients within HHAs.[50] This will allow us to estimate effect size and the intra-class correlation (ICC) required to power a future larger RCT. To measure what would have occurred with usual care (e.g., without enhanced usual care or the intervention), we will assess ED visit rates and all-cause hospitalization rates among HHAs 90 days before study onset (T=0) vs. 90 days after the time of the training course (T=1), adjusting for clustering of patients within HHAs, and stratified by trial arm.

Usage data from online materials, the mHealth app, as well as data from fidelity checklists, will be exported into excel for manual analysis by the study team.

2.13.B Analytical Plan for Qualitative Data—Interviews will be conducted and audio-recorded and professionally translated and transcribed. Data will be stored and organized in NVIVO. Using the PRISM/RE-AIM Framework, we will perform a thematic analysis, which is an approach widely used in health research when an established conceptual framework informs the topic guide and coding.[50] Two investigators trained in qualitative research methods will independently code the first three transcripts. A third investigator will review the coded transcripts and reconcile discrepancies with the team, consolidating the codes into a codebook. The codebook will be refined until all transcripts are coded. Common codes will be compared using dimensions and properties and collapsed into categories (sub-themes), which will then be further refined into broader themes.

2.13.C Sample Size Determination and Power—To detect minimal clinically important differences for both primary effectiveness outcomes (a 1-point change in mean score on the DHFKS; an 8-point change in mean score on the HF Caregiving Self-Efficacy)[43,51], with 80% power, α (two-tailed) at 0.05, and conservatively allowing for 15% attrition, we will need 104 HHAs (52 per arm). Consistent with the goals of this study (to assess feasibility and acceptability and obtain preliminary effectiveness estimates on primary outcomes), the study is not powered on patient outcomes.

3. Results

The results of this study will be disseminated to a scientific and lay audience. The study team will maintain the Clinicaltrials.gov (Identifier # NCT04239911) registration and reporting record in accordance with the WMC and Clinicaltrials.gov reporting guidelines. All data will be de-identified to maintain participant confidentiality and published in aggregate form.

Assessment of adverse events and harms

The intervention being assessed is low-risk and in line with standard clinical care at VNS Health, thus we do not anticipate any harm or adverse events. However, participants will be encouraged to report these on an ongoing basis by contacting the study team. Additionally, our study team will monitor chat logs on a weekly basis and intervene if clinically appropriate (i.e., if an HHA sends a chat to the nurse about a clinical issue and the message was not returned).

Author Manuscript Author Manuscript Author Manuscript Author Manuscript

4. Discussion

Overview of the study

This pRCT aims to leverage the role of HHAs in the care of HF patients, a high-risk and growing population with poor outcomes. With this study, we will test two main hypotheses; that an intervention aimed at training and integrating HHAs will be feasible and acceptable and that HHAs in the intervention arm will have higher HF knowledge and HF caregiving self-efficacy scores than those in the enhanced usual care arm. Finally, in an exploratory fashion, we will test the hypothesis that clinical outcomes of patients of HHAs in the intervention arm will have better outcomes compared to those in the enhanced usual care arm. The study features a partnership between an academic medical center and one of the largest home care agencies in New York, NY. The RE-AIM framework will guide implementation and evaluation and we will use a multi-method approach to data collection and analysis. The data collected from this pRCT will inform the design and delivery of a fully powered definitive trial, if deemed appropriate.

Strengths and Limitations

Strengths of our research design include a pRCT conducted in partnership with one of the largest licensed home care agencies in New York, NY, and an intervention that is focused on the HHA workforce, an integral workforce that is in frequent contact with HF patients but one which has rarely been the focus of health-related interventions. This study uses a mixed methods design, including the collection and analysis of both quantitative and qualitative data from both aides and patients as well as other key stakeholders. This is rare in home care related research and a unique opportunity to understand the experiences and impact of the trial on all involved. Finally, the intervention we are testing has been designed in partnership with HHAs and other key stakeholders in home care to meet the needs of the workforce and their patients, which offers an opportunity for future scalability.

Despite its strengths, we acknowledge several limitations of the study and its design. First, the study will be occurring during different phases of the COVID-19 pandemic, which has changed the landscape of home care, including workforce shortages of both HHAs and nurses, social distancing protocols, etc.[52] As a result, we made several changes to our approach to recruitment and onboarding, including virtual and in-person onboarding options, and the delivery of the intervention itself (e.g. broad array of healthcare professionals and staff to teach the training course, addition of text-based reminders for those in the intervention arm), and time intervals for data collection to minimize burden to participants. Second, while participants are randomly assigned to a study arm, they are not blinded to their assignment, nor are the two RAs conducting the onboarding process with them. To reduce the risk of contamination and bias, other study staff including the PI, data analysts, and statisticians are blinded, however. In line with the desire of the home health agency, our study lacks a true control group (where participants do not receive HF training); to account for this we will compare outcomes to a historical control group at the home care agency.

Author Manuscript Author Manuscript Author Manuscript Author Manuscript

Innovation and Impact

As the population ages and as more older adults with complex chronic conditions prefer to age in place, the use of home care and HHA services is increasing. Yet, most interventions aimed at improving health and healthcare delivery for high-risk patient populations have not focused on HHAs. Leveraging and enhancing the role of HHAs in HF is of particular importance given the frequent symptoms they experience, which could potentially be intervened upon with a better trained and integrated HHA workforce. This study will produce new knowledge on whether an HHA-targeted intervention is feasible, acceptable, and improves HHAs' HF knowledge and caregiver self-efficacy. If so, the results will inform a large-scale trial to be powered at the patient-level, which will use a hybrid type 1 design to test the effectiveness and implementation of the intervention on patient outcomes, including hospitalization and readmission. Finally, this study will also generate empirical data on whether such an intervention can promote workforce retention, an increasingly challenging issue in long-term care.

Supplementary Material

Refer to Web version on PubMed Central for supplementary material.

Acknowledgments

We would like to thank Ellen Percoco-Pilutis R.N., Jane Reilly R.N., Marzenka Proszoska-Lambert R.N., Valerie Hughes R.N., Michael Gelman MBA, Alana Toulopoulos MBA, Matthew Grammel BBA, and Steven Jacob for their contributions to the execution of the intervention of this study as well as to Yefrenia Henríquez Taveras, MPH, MHA, CHES for her help in the preparation of this manuscript.

Funding Sources

The research in this study was funded by the National Heart, Lung, and Blood Institute under grant number K23 HL150160. The content is solely the responsibility of the authors and does not necessarily represent the official views of the National Institutes of Health.

Research reported in this publication was also supported by the National Center for Advancing Translational Sciences of the National Institutes of Health under Award Number UL1TR002384. The content is solely the responsibility of the authors and does not necessarily represent the official views of the National Institutes of Health.

Research reported in this publication was also supported by the National Science Foundation Grant # 2026577. The content is solely the responsibility of the authors and does not necessarily represent the official views of the National Science Foundation.

References

1. Yancy CW, Jessup M, Bozkurt B, Butler J, Casey DE, Drazner MH, Fonarow GC, Geraci SA, Horwich T, Januzzi JL, Johnson MR, Kasper EK, Levy WC, Masoudi FA, McBride PE, McMurray JJ, Mitchell JE, Peterson PN, Riegel B, Sam F, Stevenson LW, Tang WH, Tsai EJ, Wilkoff BL, A.C.o.C. Foundation, A.H.A.T.F.o.P. Guidelines, 2013 ACCF/AHA guideline for the management of heart failure: a report of the American College of Cardiology Foundation/American Heart Association Task Force on Practice Guidelines, J Am Coll Cardiol 62(16) (2013) e147–239. [PubMed: 23747642]
2. Mozaffarian D, Benjamin EJ, Go AS, Arnett DK, Blaha MJ, Cushman M, Das SR, de Ferranti S, Després JP, Fullerton HJ, Howard VJ, Huffman MD, Isasi CR, Jiménez MC, Judd SE, Kissela BM, Lichtman JH, Lisabeth LD, Liu S, Mackey RH, Magid DJ, McGuire DK, Mohler ER, Moy CS, Muntner P, Mussolino ME, Nasir K, Neumar RW, Nichol G, Palaniappan L, Pandey DK, Reeves

Author Manuscript Author Manuscript Author Manuscript Author Manuscript

MJ, Rodriguez CJ, Rosamond W, Sorlie PD, Stein J, Towfighi A, Turan TN, Virani SS, Woo D, Yeh RW, Turner MB, W.G. Members, A.H.A.S. Committee, S.S. Subcommittee, Heart Disease and Stroke Statistics-2016 Update: A Report From the American Heart Association, Circulation 133(4) (2016) e38–360. [PubMed: 26673558]

3. Jencks SF, Williams MV, Coleman EA, Rehospitalizations among patients in the Medicare fee-for-service program, N Engl J Med 360(14) (2009) 1418–28. [PubMed: 19339721]
4. Krumholz HM, Merrill AR, Schone EM, Schreiner GC, Chen J, Bradley EH, Wang Y, Lin Z, Straube BM, Rapp MT, Normand SL, Drye EE, Patterns of hospital performance in acute myocardial infarction and heart failure 30-day mortality and readmission, Circ Cardiovasc Qual Outcomes 2(5) (2009) 407–13. [PubMed: 20031870]
5. Gheorghiade M, Vaduganathan M, Fonarow GC, Bonow RO, Rehospitalization for heart failure: problems and perspectives, J Am Coll Cardiol 61(4) (2013) 391–403. [PubMed: 23219302]
6. Bergethon KE, Ju C, DeVore AD, Hardy NC, Fonarow GC, Yancy CW, Heidenreich PA, Bhatt DL, Peterson ED, Hernandez AF, Trends in 30-Day Readmission Rates for Patients Hospitalized with Heart Failure: Findings from the Get with The Guidelines-Heart Failure Registry, Circ Heart Fail 9(6) (2016).
7. Chang PP, Wruck LM, Shahar E, Rossi JS, Loehr LR, Russell SD, Agarwal SK, Konety SH, Rodriguez CJ, Rosamond WD, Trends in Hospitalizations and Survival of Acute Decompensated Heart Failure in Four US Communities (2005-2014): ARIC Study Community Surveillance, Circulation 138(1) (2018) 12–24. [PubMed: 29519849]
8. Meyers AG, Salanitro A, Wallston KA, Cawthon C, Vasilevskis EE, Goggins KM, Davis CM, Rothman RL, Castel LD, Donato KM, Schnelle JF, Bell SP, Schildcrout JS, Osborn CY, Harrell FE, Kripalani S, Determinants of health after hospital discharge: rationale and design of the Vanderbilt Inpatient Cohort Study (VICS), BMC Health Serv Res 14 (2014) 10. [PubMed: 24397292]
9. Hersh AM, Masoudi FA, Allen LA, Postdischarge environment following heart failure hospitalization: expanding the view of hospital readmission, J Am Heart Assoc 2(2) (2013) e000116. [PubMed: 23580604]
10. Feltner C, Jones CD, Cené CW, Zheng ZJ, Sueta CA, Coker-Schwimmer EJ, Arvanitis M, Lohr KN, Middleton JC, Jonas DE, Transitional care interventions to prevent readmissions for persons with heart failure: a systematic review and meta-analysis, Ann Intern Med 160(11) (2014) 774–84. [PubMed: 24862840]
11. Calvillo-King L, Arnold D, Eubank KJ, Lo M, Yunyongying P, Stieglitz H, Halm EA, Impact of social factors on risk of readmission or mortality in pneumonia and heart failure: systematic review, J Gen Intern Med 28(2) (2013) 269–82. [PubMed: 23054925]
12. Sterling MR, Shaw AL, Leung PB, Safford MM, Jones CD, Tsui EK, Delgado D, Home care workers in heart failure: a systematic review, J Multidiscip Healthc 11 (2018) 481–492. [PubMed: 30288046]
13. Sterling MR, Ringel JB, Riegel B, Goyal P, Arbaje AI, Bowles KH, McDonald MV, Kern LM. Home Health Care Workers' Interactions with Medical Providers, Home Care Agencies, and Family Members for Patients with Heart Failure. J Am Board Fam Med. 2023 Apr 3;36(2):369–375. doi:10.3122/jabfm.2022.220204R2. Epub 2023 Mar 22. [PubMed: 36948539]
14. Cheng RK, Cox M, Neely ML, Heidenreich PA, Bhatt DL, Eapen ZJ, Hernandez AF, Butler J, Yancy CW, Fonarow GC, Outcomes in patients with heart failure with preserved, borderline, and reduced ejection fraction in the Medicare population, Am Heart J 168(5) (2014) 721–30. [PubMed: 25440801]
15. Arundel C, Lam PH, Khosla R, Blackman MR, Fonarow GC, Morgan C, Zeng Q, Fletcher RD, Butler J, Wu WC, Deedwania P, Love TE, White M, Aronow WS, Anker SD, Allman RM, Ahmed A, Association of 30-Day All-Cause Readmission with Long-Term Outcomes in Hospitalized Older Medicare Beneficiaries with Heart Failure, Am J Med 129(11) (2016) 1178–1184. [PubMed: 27401949]
16. Ziaeian B, Fonarow GC, The Prevention of Hospital Readmissions in Heart Failure, Prog Cardiovasc Dis 58(4) (2016) 379–85. [PubMed: 26432556]
17. Barnett S, Jones SC, Bennett S, Iverson D, Bonney A, Perceptions of family physician trainees and trainers regarding the usefulness of a virtual community of practice, J Med Internet Res 15(5) (2013) e92. [PubMed: 23666237]

Author Manuscript Author Manuscript Author Manuscript Author Manuscript

18. PHI, Direct Care Workers in the United States: Key Facts 2022, Public Health Institute, September 2022.
19. Jones CD, Ginde AA, Burke RE, Wald HL, Masoudi FA, Boxer RS, Increasing Home Healthcare Referrals upon Discharge from U.S. Hospitals: 2001-2012, J Am Geriatr Soc 63(6) (2015) 1265–6. [PubMed: 26096410]
20. Jones CD, Wald HL, Boxer RS, Masoudi FA, Burke RE, Capp R, Coleman EA, Ginde AA, Characteristics Associated with Home Health Care Referrals at Hospital Discharge: Results from the 2012 National Inpatient Sample, Health Serv Res 52(2) (2017) 879–894. [PubMed: 27196526]
21. Bercovitz A, Moss A, Sengupta M, Park-Lee EY, Jones A, Harris-Kojetin LD, An overview of home health aides: United States, 2007, Natl Health Stat Report (34) (2011) 1–31.
22. Hewko SJ, Cooper SL, Huynh H, Spiwek TL, Carleton HL, Reid S, Cummings GG, Invisible no more: a scoping review of the health care aide workforce literature, BMC Nurs 14 (2015) 38. [PubMed: 26203297]
23. Sterling MR, Silva AF, Leung PBK, Shaw AL, Tsui EK, Jones CD, Robbins L, Escamilla Y, Lee A, Wiggins F, Sadler F, Shapiro MF, Charlson ME, Kern LM, Safford MM, "It's Like They Forget That the Word 'Health' Is in 'Home Health Aide'": Understanding the Perspectives of Home Care Workers Who Care for Adults With Heart Failure, J Am Heart Assoc 7(23) (2018) e010134. [PubMed: 30571599]
24. Sterling MR, Silva AF, Robbins L, Dargar SK, Schapira MM, Safford MM, Let's talk numbers: a qualitative study of community-dwelling US adults to understand the role of numeracy in the management of heart failure, BMJ Open 8(9) (2018) e023073.
25. Russell D, Mola A, Onorato N, et al. Preparing Home Health Aides to Serve as Health Coaches for Home Care Patients with Chronic Illness: Findings and Lessons Learned from a Mixed-Method Evaluation of Two Pilot Programs. Home Health Care Management & Practice. 2017;29(3):191–198. doi:10.1177/1084822317706080.
26. Sterling MR, Barbaranelli C, Riegel B, Stawnychy M, Ringel JB, Cho J, Vellone E, The Influence of Preparedness, Mutuality, and Self-efficacy on Home Care Workers' Contribution to Self-care in Heart Failure: A Structural Equation Modeling Analysis, J Cardiovasc Nurs 37(2) (2022) 146–157. [PubMed: 33315614]
27. Feldman PH, Barrón Y, Onorato N, Russell D, Sterling MR, McDonald M. Covid-19: Home Health Aides' Perceived Preparedness and Self-Reported Availability for Work: Six Month Survey Results. New Solut. 2023 Nov;33(2-3):130–148. doi: 10.1177/10482911231199449. Epub 2023 Sep 5. [PubMed: 37670604]
28. Sterling MR, Cho J, Ringel JB, Avgar AC, Heart Failure Training and Job Satisfaction: A Survey of Home Care Workers Caring for Adults with Heart Failure in New York City, Ethn Dis 30(4) (2020) 575–582. [PubMed: 32989357]
29. Okeke F, Tseng E, Piantella B, Brown M, Kaur H, Sterling MR, & Dell N Technology, home health care, and heart failure: a qualitative analysis with multiple stakeholders. Proceedings of the 2nd ACM SIGCAS Conference on Computing and Sustainable Societies. 2019
30. Tseng E, Okeke F, Sterling M, Dell N "We can learn. Why not?": Designing Technologies to Engender Equity for Home Health Aides. *In* Proceedings of the 2020 CHI Conference on Human Factors in Computing Systems (CHI '20). Association for Computing Machinery, New York, NY, USA, 1–14. 10.1145/3313831.3376633
31. Sterling MR et al., 911 Calls by HCWs, Manuscript in Preparation, 2023
32. Leung PB, Silva AF, Cho J, Kaur H, Lee A, Escamilla Y, Wiggins F, Safford MM, Kern LM, Shalev A, Sterling MR, Eliciting the educational priorities of home care workers caring for adults with heart failure, Gerontol Geriatr Educ 43(2) (2022) 239–249. [PubMed: 32666906]
33. Sterling MR, Cho J, Leung PB, Silva AF, Ringel J, Wiggins F, Herring N, Powell A, Toro O, Lee A, Loughman J, Obodai M, Poon A, Goyal P, Kern LM, Safford MM. Development and Piloting of a Community-Partnered Heart Failure Training Course for Home Health Care Workers. Circ Cardiovasc Qual Outcomes. 2022 Nov;15(11): e009150. doi: 10.1161/CIRCOUTCOMES.122.009150. Epub 2022 Nov 15. [PubMed: 36378772]
34. VNS Health, “Who We Are”, VNS Health, https://www.vnshealth.org/about/, 2018.
35. CareConnect Mobile, “CareConnect”, https://www.careconnectmobile.com/, 2023

Author Manuscript Author Manuscript Author Manuscript Author Manuscript

36. Sterling MR, Dell N, Piantella B, Cho J, Kaur H, Tseng E, Okeke F, Brown M, Leung PBK, Silva AF, Shaw AL, Kern LM. Understanding the Workflow of Home Health Care for Patients with Heart Failure: Challenges and Opportunities. J Gen Intern Med. 2020 Jun;35(6):1721–1729. doi: 10.1007/s11606-020-05675-8. [PubMed: 32026253]
37. Sterling MR, Dell N, Tseng E, Okeke F, Cho J, Piantella B, Tobin JN. Home care workers caring for adults with heart failure need better access to training and technology: A role for implementation science. J Clin Transl Sci. 2020 Apr 6;4(3):224–228. doi: 10.1017/cts.2020.36. [PubMed: 32695493]
38. Leung PB, Silva AF, Cho J, Kaur H, Lee A, Escamilla Y, Wiggins F, Safford MM, Kern LM, Shalev A Sterling MR The Development of a Training Course for Home Care Workers caring for adults with heart failure. Journal of General Internal Medicine. July 2020. Vol. 35, No. SUPPL 1, pp. S773–S773). SPRINGER.
39. Carmel AS, Cornelius-Schecter A, Frankel B, Jannat-Khah D, Sinha S, Pelzman F, Safford MM. Evaluation of the Patient Activated Learning System (PALS) to improve knowledge acquisition, retention, and medication decision making among hypertensive adults: Results of a pilot randomized controlled trial. Patient Educ Couns. 2019 Aug;102(8):1467–1474. doi: 10.1016/j.pec.2019.03.001. Epub 2019 Mar 4. [PubMed: 30928344]
40. Glasgow RE, Vogt TM, Boles SM, Evaluating the public health impact of health promotion interventions: the RE-AIM framework, Am J Public Health 89(9) (1999) 1322–7. [PubMed: 10474547]
41. Harden SM, Smith ML, Ory MG, Smith-Ray RL, Estabrooks PA, Glasgow RE, RE-AIM in Clinical, Community, and Corporate Settings: Perspectives, Strategies, and Recommendations to Enhance Public Health Impact, Front Public Health 6 (2018) 71. [PubMed: 29623270]
42. Kessler RS, Purcell EP, Glasgow RE, Klesges LM, Benkeser RM, Peek CJ, what does it mean to "employ" the RE-AIM model? Eval Health Prof 36(1) (2013) 44–66. [PubMed: 22615498]
43. van der Wal MH, Jaarsma T, Moser DK, van Veldhuisen DJ, Development and testing of the Dutch Heart Failure Knowledge Scale, Eur J Cardiovasc Nurs 4(4) (2005) 273–7. [PubMed: 16126459]
44. Vellone E, Barbaranelli C, Pucciarelli G, Zeffiro V, Alvaro R, Riegel B, Validity and Reliability of the Caregiver Contribution to Self-Care of Heart Failure Index Version 2, J Cardiovasc Nurs 35(3) (2020) 280–290. [PubMed: 32084084]
45. Riegel B, Lee CS, Dickson VV, Carlson B, An update on the self-care of heart failure index, J Cardiovasc Nurs 24(6) (2009) 485–97. [PubMed: 19786884]
46. Gagne M and Berube N and Donia M and Houfort N and Lvina E 2016. Validation evidence for the Work Domain Satisfaction Scale in two languages. International Journal of Psychological Studies. 8 (3): pp. 26–39. 10.5539/ijps.v8n3p26
47. Bothma CFC, & Roodt G (2013). The validation of the turnover intention scale. SA Journal of Human Resource Management/SA Tydskrif vir Menslikehulpbronbestuur, 11(1), Art. #507, 12 pages. 10.4102/sajhrm.v11i1.507
48. Spertus JA, Jones PG. Development and Validation of a Short Version of the Kansas City Cardiomyopathy Questionnaire. Circ Cardiovasc Qual Outcomes. 2015 Sep;8(5):469–76. doi: 10.1161/CIRCOUTCOMES.115.001958. [PubMed: 26307129]
49. West Brady T. W KB, Galecki Andrzej T.. Linear Mixed Models: A Practical Guide Using Statistical Software. Second Edition ed: Chapman and Hall/CRC; 2014.
50. Terry G, Hayfield N, Clarke V, & Braun V (2017). Thematic analysis. In Willig C, & Rogers W. Stainton (Eds.), The SAGE Handbook of Qualitative Research in Psychology (17–37). (2nd). London: SAGE Publications
51. Vellone E, Rebora P, Iovino P, Ghizzardi G, Baricchi M, Alvaro R, Sili A, Barello S, Ausili D, Trenta AM, Pedroni C, Dellafiore F, Arrigoni C, Riegel B, Caruso R. Remote motivational interviewing to improve patient self-care and caregiver contribution to self-care in heart failure (REMOTIVATE-HF): Rationale, design, and methodology for a multicentre randomized controlled trial. Res Nurs Health. 2023 Apr;46(2):190–202. doi: 10.1002/nur.22289. Epub 2022 Dec 25. [PubMed: 36566360]

Author Manuscript Author Manuscript Author Manuscript Author Manuscript

52. Sterling MR, Tseng E, Poon A, Cho J, Avgar AC, Kern LM, Ankuda CK, Dell N, Experiences of Home Health Care Workers in New York City During the Coronavirus Disease 2019 Pandemic: A Qualitative Analysis, JAMA Intern Med 180(11) (2020) 1453–1459. [PubMed: 32749450]

Author Manuscript Author Manuscript Author Manuscript Author Manuscript

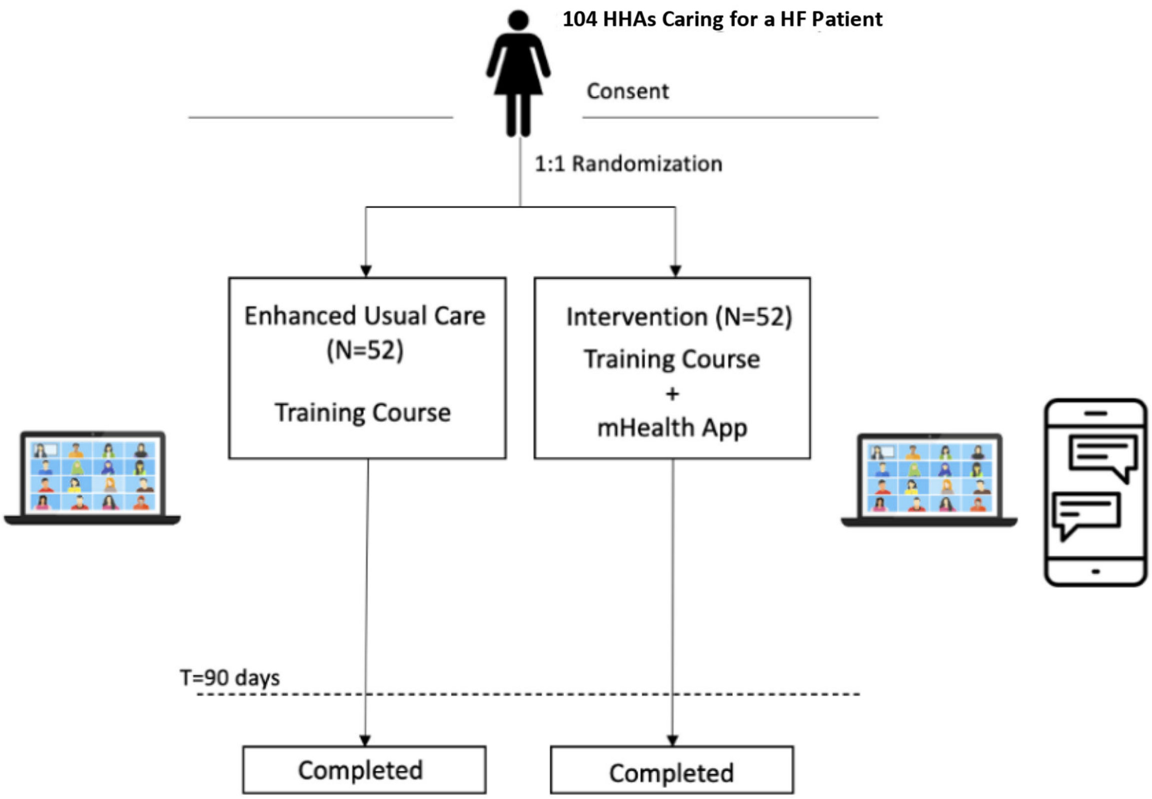

Figure 1.
Schematic of Pilot RCT with HHAs caring for HF Patients

Author Manuscript Author Manuscript Author Manuscript Author Manuscript

Table 1.

Summary of Participant Timeline and Data Collection Points

Participant and Type of Data Collected	T_0 Baseline	T_1 Post HF Course*	T_2 45 Days**	T_3 90 Days
Home health aides and attendants (HHA)				
**HHA Characteristics (Survey)**				
Sociodemographic Data (Sex, Age, Race, Ethnicity, Highest Education Level, Primary language spoken at home)<br><br>Employment Data (Years on the job, number of agencies worked for previously, Years at current agency, Number of HF clients cared for, Average hours per week spent with HF patient, Previous training regarding HF)	X			
**Feasibility and Acceptability Measures (Surveys, Interviews, Usage data)**				
Course Satisfaction and Engagement (Survey)		X		
General Experiences in Study, HF training, patients (Both Arms)- (Interview)			X	X
Experiences with Chat feature, nurses, patients (Intervention arm only) (Interview)			X	X
Use of online educational materials (both arms); Use of chat feature (intervention arm)				X
**Primary Outcomes (Survey)**				
HF Knowledge (DHFKS)	X	X		X
HF Caregiving Self-Efficacy (CC-SCHFI)	X	X		X
**Secondary Outcomes (Survey)**				
Intention to Leave (Turnover Intention Scale)	X			X
Job Satisfaction (Work Domain Satisfaction Scale)	X			
Communication (Survey/Interview)	X		X	X
Patient				
**Feasibility and Acceptability (Interview)**				X
**Exploratory Outcomes*** **				
Emergency Department Visit (All-Cause) [Service Use Data]				X
Functional Status (Service Use Data)				X
Hospitalization (Service Use Data)				X
Quality of Life (Cardiomyopathy Questionnaire [Kansas City]) {Interview}				X

* Within 24 hours of HF training course

** Post-HF Training Course

*** Will be ascertained during a 90-day, pre-study period, for comparison.

Author Manuscript Author Manuscript Author Manuscript Author Manuscript

Table 2.

RE-AIM Evaluation of Study Outcomes and Design

Dimensions	Definition	In the Context of our Study	Data source
Reach	The absolute number, proportion, and representativeness of individuals willing to participate in the study	• Eligibility Criteria<br>• Eligible target population<br>• Number of HHAs approached<br>• Number of HHAs eligible but did not participate<br>• Barriers to recruitment	• Research protocol<br>• Screening form<br>• Records (data recruitment) from study staff<br>• Demographic survey and interview question
Effectiveness	The impact of an intervention on outcomes (primary, secondary, exploratory)	Primary outcomes: HHA Level<br>• HF Knowledge<br>• HF Caregiving Self-Efficacy<br>• Job Satisfaction<br>• Intention to Leave<br>Exploratory outcomes: Patient Level<br>• Hospitalization<br>• ED visit<br>• Readmission<br>– Quality of Life	• Surveys<br>• Interviews<br>• Claims data (service use)
Adoption	The absolute number, proportion, and representativeness of participants who participate in the study	Staff (RAs, RNs,)<br>• Roles<br>• Credentials<br>• Experience, including motivating factors and barriers<br>HHAs<br>• Number approached<br>• Number who completed onboarding, training, trial<br>• Extent to which the intervention was adopted	• Research records<br>• Attendance records<br>• Interviews
Implementation	The study and interventions' fidelity to the protocol, including consistency of delivery as intended, time, and interventions' adaptations	• Feasibility<br>• Acceptability<br>• Fidelity to study protocol by staff, course instructors, and RNs<br>• Adaptations to the intervention<br>• Use of intervention materials (online training, chats)<br>• Study teams' deliver of the intervention<br>• Challenges/barriers to delivery/ implementation	• Research record<br>• Observations by study team<br>• Surveys<br>• Interviews<br>• Data from CareConnect<br>• Data from Google analytics<br>• Interviews with study staff<br>• Interviews with participants

Dimensions	Definition	In the Context of our Study	Data source
		• Successes	• Interviews with agency staff and leadership
Maintenance	The extent to which the intervention becomes part of the routine organizational practices and policies	• To be assessed in future large-scale study. (Although we can look at usage of materials [online] and if chats occur after 90 days)	

Author Manuscript Author Manuscript Author Manuscript Author Manuscript
