# Supplementary material for: Home Health Aides Caring for Adults With Heart Failure: A Pilot Randomized Clinical Trial
Source: JAMA Netw Open. 2025 Nov 10;8(11):e2548121. doi: 10.1001/jamanetworkopen.2025.48121 (PMC12603854; doi:10.1001/jamanetworkopen.2025.48121)
Supplement: Supplement 2. — eFigure 1. Model based Outcome Trajectory by Arm Among Participants with Low Baseline score eFigure 2. Exclusion cascade for the analysis of patient outcomes eTable 1. Participant (Home health aide) characteristics by baseline DHFKS eTable 2. Participant (Home health aide) characteristics by baseline Self-efficacy [file jamanetwopen-e2548121-s002.pdf]

Supplemental Online Content

Sterling MR, Espinosa CG, Vergez S, et al. Home health aides caring for adults with heart failure: a pilot randomized clinical trial. *JAMA Netw Open*. 2025;8(11):e2548121. doi:10.1001/jamanetworkopen.2025.48121

eFigure 1. Model based Outcome Trajectory by Arm Among Participants with Low Baseline score

eFigure 2. Exclusion cascade for the analysis of patient outcomes

eTable 1. Participant (Home health aide) characteristics by baseline DHFKS

eTable 2. Participant (Home health aide) characteristics by baseline Self-efficacy

This supplemental material has been provided by the authors to give readers additional information about their work.

© 2025 Sterling MR et al. *JAMA Network Open*.

eSupplemental Figure 1. Model based Outcome Trajectory by Arm Among Participants with Low Baseline score

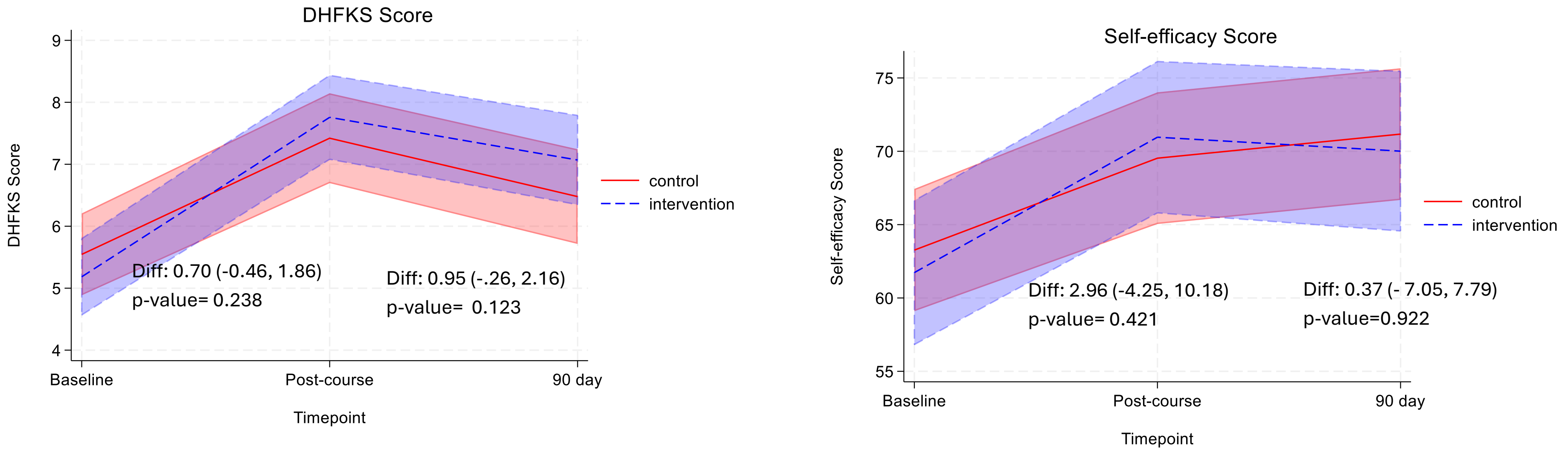

Dutch HF Knowledge Scale (DHFKS), a 15-item scale which measures HF knowledge including treatments and symptom recognition. Scores range from 0-15, with higher scores indicating higher knowledge.

HF caregiving self-efficacy is a 10 item sub-scale of Caregiver Contribution to Self-Care in HF Index (CC-SCHFI). HF caregiving self-efficacy scores range from 0-100, with higher scores indicating greater self-efficacy.

Low baseline scores for each measure were determined by using median cut-point of baseline values

Mean values and differences were calculated from mixed effects models that included a fixed effects categorical variable for time point (baseline/post-course/90 day), an indicator for study arm (control/intervention), a study arm by timepoint interaction and subject-specific random intercept.

© 2025 Sterling MR et al. *JAMA Network Open*.

eSupplemental Figure 2. Exclusion cascade for the analysis of patient outcomes

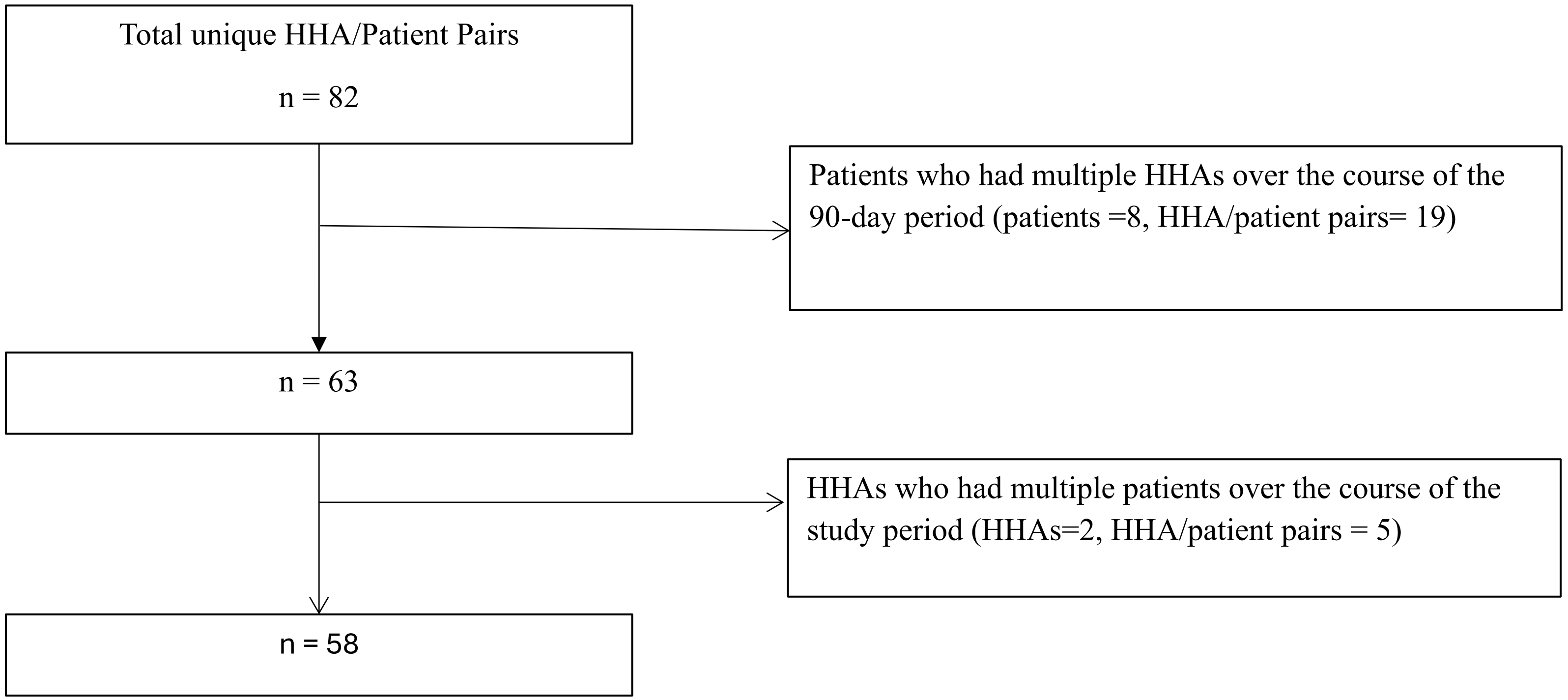

There were multiple cases of study HHA's assigned to multiple patients. Additionally, there were multiple cases of patients who were assigned to multiple HHA's participating in the study. These cases were removed from the analysis to reduce complexity.

© 2025 Sterling MR et al. *JAMA Network Open*.

eSupplemental Table 1. Participant (Home health aide) characteristics by baseline DHFKS

Characteristics	***Low Baseline DHFKS***	***High Baseline DHFKS***	***p-value***
N	71	31	
Sex			0.58
M	2 (3%)	2 (6%)	
F	69 (97%)	29 (94%)	
Age, mean (SD)	54.6 (10.7)	52.6 (10.2)	0.39
Race			0.13
African American, Black	38 (55%)	24 (77%)	
American Indian/Alaska Native	1 (1%)	0 (0%)	
Asian	7 (10%)	0 (0%)	
White	6 (9%)	3 (10%)	
Other	17 (25%)	4 (13%)	
Hispanic			0.50
Yes	20 (29%)	7 (23%)	
No	49 (71%)	24 (77%)	
Education			0.42
No degree or some high school	14 (20%)	7 (23%)	
Completed high school or GED	23 (33%)	13 (42%)	
Some college	14 (20%)	6 (19%)	
College degree	15 (21%)	2 (6%)	
Graduate degree	4 (6%)	3 (10%)	
Born in the US?			0.87
Yes	10 (14%)	4 (13%)	
No	61 (86%)	27 (87%)	
What is your primary language?			0.38
English	45 (63%)	20 (65%)	
Spanish	16 (23%)	4 (13%)	
Other	10 (14%)	7 (23%)	
Years as home care aide			0.16
0-5	23 (32%)	5 (16%)	

© 2025 Sterling MR et al. *JAMA Network Open*.

6-10	22 (31%)	9 (29%)	
11-15	8 (11%)	8 (26%)	
>15	18 (25%)	9 (29%)	
How many home care agencies worked for in the past			0.70
0-1	37 (54%)	14 (45%)	
2	22 (32%)	11 (35%)	
>=3	10 (14%)	6 (19%)	
Years worked at current agency			0.030
0-5	29 (41%)	6 (19%)	
6-10	25 (36%)	9 (29%)	
11-15	9 (13%)	9 (29%)	
>15	7 (10%)	7 (23%)	
Number of heart failure patients cared for			0.67
<=5	46 (65%)	19 (61%)	
>5	11 (15%)	7 (23%)	
Not Sure	14 (20%)	5 (16%)	
Hours per week spent with heart failure client			0.60
1-5	9 (13%)	6 (19%)	
6-10	24 (35%)	10 (32%)	
11-20	8 (12%)	4 (13%)	
>20	16 (23%)	9 (29%)	
Not Sure	12 (17%)	2 (6%)	
Heart failure training			0.56
None	31 (44%)	15 (48%)	
A little	24 (34%)	7 (23%)	
Some	15 (21%)	8 (26%)	
A lot	1 (1%)	1 (3%)	
Management (CC-SCHFI), median (IQR)	55.0 (37.5, 67.5)	60.0 (50.0, 75.0)	0.084
Maintenance (CC-SCHFI), median (IQR)	85.0 (76.7, 93.3)	86.7 (76.7, 96.7)	0.18

© 2025 Sterling MR et al. *JAMA Network Open*.

eSupplemental Table 2. Participant (Home health aide) characteristics by baseline Self-efficacy

Characteristics	*Low Baseline Self-Efficacy*	*High Baseline Self-Efficacy*	*p-value*
N	62	39	
Sex			0.16
M	4 (6%)	0 (0%)	
F	58 (94%)	39 (100%)	
Age, mean (SD)	53.6 (10.9)	54.7 (10.2)	0.61
Race			0.44
African American, Black	40 (65%)	21 (57%)	
American Indian/Alaska Native	0 (0%)	1 (3%)	
Asian	4 (6%)	3 (8%)	
White	7 (11%)	2 (5%)	
Other	11 (18%)	10 (27%)	
Hispanic			0.67
Yes	16 (26%)	11 (30%)	
No	46 (74%)	26 (70%)	
education			0.047
No degree or some high school	14 (23%)	7 (18%)	
Completed high school or GED	21 (34%)	14 (36%)	
Some college	13 (21%)	7 (18%)	
College degree	6 (10%)	11 (28%)	
Graduate degree	7 (11%)	0 (0%)	
Born in the US?			0.81
Yes	9 (15%)	5 (13%)	
No	53 (85%)	34 (87%)	
What is your primary language?			0.14
English	36 (58%)	28 (72%)	
Spanish	12 (19%)	8 (21%)	
Other	14 (23%)	3 (8%)	
Years as home care aide			0.48

© 2025 Sterling MR et al. *JAMA Network Open*.

0-5	20 (32%)	8 (21%)	
6-10	19 (31%)	11 (28%)	
11-15	9 (15%)	7 (18%)	
>15	14 (23%)	13 (33%)	
How many home care agencies worked for in the past			0.43
0-1	34 (56%)	17 (44%)	
2	19 (31%)	14 (36%)	
>=3	8 (13%)	8 (21%)	
Years worked at current agency			0.79
0-5	23 (38%)	12 (31%)	
6-10	20 (33%)	13 (33%)	
11-15	11 (18%)	7 (18%)	
>15	7 (11%)	7 (18%)	
Number of heart failure patients cared for			0.55
<=5	41 (66%)	23 (59%)	
>5	9 (15%)	9 (23%)	
Not Sure	12 (19%)	7 (18%)	
Hours per week spent with heart failure client			0.77
1-5	11 (18%)	4 (10%)	
6-10	19 (31%)	15 (38%)	
11-20	8 (13%)	4 (10%)	
>20	14 (23%)	11 (28%)	
Not Sure	9 (15%)	5 (13%)	
Heart failure training			0.015
None	34 (55%)	12 (31%)	
A little	19 (31%)	12 (31%)	
Some	9 (15%)	13 (33%)	
A lot	0 (0%)	2 (5%)	
Management (CC-SCHFI), median (IQR)	50.0 (40.0, 60.0)	65.0 (45.0, 75.0)	0.009
Maintenance (CC-SCHFI), median (IQR)	83.3 (76.7, 93.3)	86.7 (76.7, 93.3)	0.25

© 2025 Sterling MR et al. *JAMA Network Open*.
