# Supplementary material for: Home Health Aides Caring for Adults With Heart Failure: A Pilot Randomized Clinical Trial
Source: JAMA Netw Open. 2025 Nov 10;8(11):e2548121. doi: 10.1001/jamanetworkopen.2025.48121 (PMC12603854; doi:10.1001/jamanetworkopen.2025.48121)
Supplement: Supplement 3. — Data Sharing Statement [file jamanetwopen-e2548121-s003.pdf]

Data Sharing Statement

Sterling. Home Health Aides Caring for Adults With Heart Failure. *JAMA Netw Open*. Published November 10, 2025. doi:10.1001/jamanetworkopen.2025.48121

Data
Additional Information: NCT04239911
Data available: Yes
Data types: Data dictionary, Other (please specify)
Additional Information: statistical/analytic dode, qualitative code book
How to access data: mrs9012@med.cornell.edu
When available: With publication

Supporting Documents
Document types: Statistical/analytic code
How to access documents: mrs9012@med.cornell.edu
When available: With publication

Additional Information
Who can access the data: researchers whose proposed use of the data has been approved
Types of analyses: specified purpose
Mechanisms of data availability: approval of proposal
